# Engineered Fluorescent Strains of Cryptococcus neoformans: a Versatile Toolbox for Studies of Host-Pathogen Interactions and Fungal Biology, Including the Viable but Nonculturable State

**DOI:** 10.1128/spectrum.01504-22

**Published:** 2022-08-25

**Authors:** Raffael Júnio Araújo de Castro, Marco Túlio Aidar Mariano Rêgo, Fabiana S. Brandão, Ana Laura Alfonso Pérez, Janice Lisboa De Marco, Marcio José Poças-Fonseca, Connie Nichols, J. Andrew Alspaugh, Maria Sueli S. Felipe, Alexandre Alanio, Anamélia Lorenzetti Bocca, Larissa Fernandes

**Affiliations:** a Laboratory of Applied Immunology, Campus Darcy Ribeiro, University of Brasília, Asa Norte, Brasília, Federal District, Brazil; b Faculty of Health Science, Campus Darcy Ribeiro, University of Brasília, Asa Norte, Brasília, Federal District, Brazil; c Department of Cell Biology, Institute of Biological Sciences, Campus Darcy Ribeiro, University of Brasília, Asa Norte, Brasilia, Federal District, Brazil; d Department of Genetics and Morphology, Institute of Biological Sciences, Campus Darcy Ribeiro, University of Brasília, Asa Norte, Brasília, Federal District, Brazil; e Duke University School of Medicine, Department of Medicine, Durham, North Carolina, USA; f Catholic University of Brasilia, Campus Asa Norte, Asa Norte, Brasília, Federal District, Brazil; g CNRS, Unité de Mycologie Moléculaire, Centre National de Référence Mycoses et Antifongiques, Institut Pasteur, Paris, France; h Laboratoire de Mycologie et Parasitologie, AP-HP, Hôpital Saint Louis, Université Paris Diderot, Sorbonne Paris Cité, Paris, France; i Faculty of Ceilândia, Campus UnB Ceilândia, University of Brasília, Ceilândia Sul, Brasília, Federal District, Brazil; University of Minnesota Medical School

**Keywords:** *Cryptococcus neoformans*, fluorescently tagged strains, H99, poly(A) binding protein, histone H3, GFP, mCherry, VBNC, dormancy, stress granules

## Abstract

Cryptococcus neoformans is an opportunistic fungal pathogen known for its remarkable ability to infect and subvert phagocytes. This ability provides survival and persistence within the host and relies on phenotypic plasticity. The viable but nonculturable (VBNC) phenotype was recently described in C. neoformans, whose study is promising in understanding the pathophysiology of cryptococcosis. The use of fluorescent strains is improving host interaction research, but it is still underexploited. Here, we fused histone H3 or the poly(A) binding protein (Pab) to enhanced green fluorescent protein (eGFP) or mCherry, obtaining a set of C. neoformans transformants with different colors, patterns of fluorescence, and selective markers (hygromycin B resistance [Hyg^r^] or neomycin resistance [Neo^r^]). We validated their similarity to the parental strain in the stress response, the expression of virulence-related phenotypes, mating, virulence in Galleria mellonella, and survival within murine macrophages. PAB-GFP, the brightest transformant, was successfully applied for the analysis of phagocytosis by flow cytometry and fluorescence microscopy. Moreover, we demonstrated that an engineered fluorescent strain of C. neoformans was able to generate VBNC cells. GFP-tagged Pab1, a key regulator of the stress response, evidenced nuclear retention of Pab1 and the assembly of cytoplasmic stress granules, unveiling posttranscriptional mechanisms associated with dormant C. neoformans cells. Our results support that the PAB-GFP strain is a useful tool for research on C. neoformans.

**IMPORTANCE** Cryptococcus neoformans is a human-pathogenic yeast that can undergo a dormant state and is responsible for over 180,000 deaths annually worldwide. We engineered a set of fluorescent transformants to aid in research on C. neoformans. A mutant with GFP-tagged Pab1 improved fluorescence-based techniques used in host interaction studies. Moreover, this mutant induced a viable but nonculturable phenotype and uncovered posttranscriptional mechanisms associated with dormant C. neoformans. The experimental use of fluorescent mutants may shed light on C. neoformans-host interactions and fungal biology, including dormant phenotypes.

## INTRODUCTION

The incidence of fungal infections has increased considerably in recent decades, becoming a notable worldwide public health problem with high rates of mortality in patients living with HIV/AIDS (1, 2). Cryptococcus neoformans stands out among opportunistic pathogens, with a mortality rate of up to 60% among HIV patients (3, 4). C. neoformans desiccated yeast cells or spores are frequently inhaled, causing a primary infection normally contained in the immunocompetent host (5). This latent infection becomes active under immunocompromised conditions, followed by fungal dissemination to the central nervous system causing meningoencephalitis, which is fatal in the absence of early diagnosis and treatment (6, 7). Recent studies point to about 5,300 cases and 2,400 deaths per year only in Latin America. The worldwide estimate is around 220,000 cases annually, over 180,000 of which result in patient death (3, 8).

C. neoformans is a facultative intracellular pathogen known for its remarkable ability to evade and subvert the immune response, mainly by manipulating phagocytes, key players in anticryptococcal defense (9). This ability relies on the expression of a set of virulence determinants and phenotypic plasticity that support survival and persistence within the host (10). The major virulence determinants consist of the formation of the polysaccharide capsule, which is composed mainly of glucuronoxylomannan (GXM) and glucuronoxylomannogalactan (GXMgal) (11), conferring antiphagocytic and immunomodulatory properties (12), and the production of melanin, which provides protection against several stressors such as heat and oxidative radicals (10). Other virulence traits that influence the success of C. neoformans infection and dissemination include the production of the secreted enzymes phospholipase and urease, which are involved in intracellular replication, escape from phagocytes, as well as brain invasion (13–15).

Despite being found predominantly in the yeast form, C. neoformans also produces hyphae and spores during sexual reproduction. Although spores of two distinct mating types (MAT**a** and MATα) are produced during the mating process, MATα spores predominate among clinical isolates and have greater tropism for the central nervous system and virulence potential (16–18).

A recently described C. neoformans physiological state, the viable but nonculturable (VBNC) phenotype, describes a nonreplicative state of microbial dormancy featuring low metabolic activity and a loss of culturability on routine agar media. Dormant cells, including VBNC cells, can be induced under stress conditions such as nutrient and oxygen deprivation, enhancing the chances of survival and persistence until conditions are more permissive for regrowth (19). In C. neoformans, dormant cells were first described during experimental murine infection and interactions with macrophages *in vitro* (20). More recently, a method for the *in vitro* generation of VBNC C. neoformans cells was implemented by Hommel and colleagues, allowing better characterization and understanding of these cells, including growth latency, decreased global metabolism but increased mitochondrial expression, and reactivation upon the addition of pantothenic acid stimuli (21). Nevertheless, little is known about the biology of VBNC cells (22).

Fluorescent strains have been increasingly used as valuable tools to monitor the dynamics of fungal infections and the progression of the disease as well as to gather information on the host response and the therapeutic efficacy of antifungal agents (23). These engineered strains can be used for a wide variety of specific experimental purposes, such as in the assessment of gene expression during infection (24); analyses of fungal viability (25), biofilm formation (26), genome stability (27), and cell cycle dynamics (28); quantification of fungal loads *in vitro* and *in vivo* (25, 29, 30); and functional studies related to fungal reproduction (31). Other interesting applications include fluorescent reporters as proxies for environmental conditions such as pH (32) and carbon source and oxygen availability (33).

In the present work, we engineered the widely used C. neoformans H99 strain to express green fluorescent protein (GFP) or mCherry fused to histone H3 to target fluorophores to the nuclear compartment or poly(A) binding protein (Pab) to direct fluorophores to the cytoplasm, using different dominant selective markers, hygromycin B resistance (Hyg^r^) or neomycin resistance (Neo^r^). We applied several tests to rule out functional divergences from the wild type (WT): evaluations of growth in response to stress, the expression of virulence-related phenotypes, mating capacity, survival within murine macrophages, and virulence in the invertebrate model Galleria mellonella. PAB-GFP was successfully employed in a fluorescence-based analysis of C. neoformans-host interactions as well as in the generation of VBNC yeast cells. PAB-GFP VBNC cells maintained the GFP signal, which in turn demonstrated a substantively altered intracellular distribution pattern of Pab1 localization, suggesting previously undescribed posttranscriptional processes associated with the dormant state of C. neoformans.

## RESULTS

### Construction and validation of GFP- or mCherry-fluorescent strains.

The strategy used in our study for obtaining fluorescent mutants of C. neoformans consisted of the construction of fluorescent proteins fused to proteins with well-defined subcellular localizations. Based on this approach, we created expression alleles using the *H3* and *PAB* genes from the genome of H99 fused to the genes encoding the fluorescent protein enhanced green fluorescent protein (eGFP) or mCherry. *H3* codes for the highly conserved histone H3 DNA binding protein that plays an important role in the regulation of chromatin structure and gene expression (34). The product of the *PAB1* gene [poly(A) binding protein] binds to the poly(A) tail of the cytoplasmic mRNA and has a central function in the regulation of gene expression in eukaryotes (35). The choice of these genes was due to the distinct cytolocations of their products, which allows different patterns of cell labeling: histone H3 localizes to the nucleus, while Pab1 is enriched in the cytoplasm (34, 36).

In order to expand the Cryptococcus molecular biology toolbox, we created fluorescent mutants carrying different selective antibiotic markers. The constructs containing fusions with eGFP were cloned into the pSDMA57 plasmid (37), which carries a neomycin resistance gene (see Fig. S1 and S2 in the supplemental material). The constructs with the red fluorescent protein mCherry were inserted into pSDMA58 (37), which contains the gene for resistance to hygromycin B (Fig. S1 and S2). Strains transformed with pSDMA57 or pSDMA58 carrying GFP or mCherry not fused to any gene were considered negative controls (Ø GFP and Ø mCherry, respectively). Based on previous work, the mutant alleles were targeted to a small intergenic region called the “safe haven” (SH), in which insertions appear not to compromise the expression of neighboring genes (37). In our study, we selected the mutants based on high levels of fluorescent protein expression and the expected patterns of subcellular localization. The transformation of selected mutants was confirmed by PCR and Western blot analysis (Fig. S3 and S4). Of note, along with the robust expression of fused PAB-GFP, a small amount of free GFP (~27-kDa band) was also detected in PAB-GFP transformants by Western blotting and probably resulted from the posttranslational proteolysis of the PAB-GFP fusion protein (Fig. S4). We also verified by PCR if the constructs were inserted into the safe haven (Fig. S5); however, we characterized our C. neoformans fluorescent mutants regardless of whether the cassette had entered this site of the genome.

**FIG 1:**
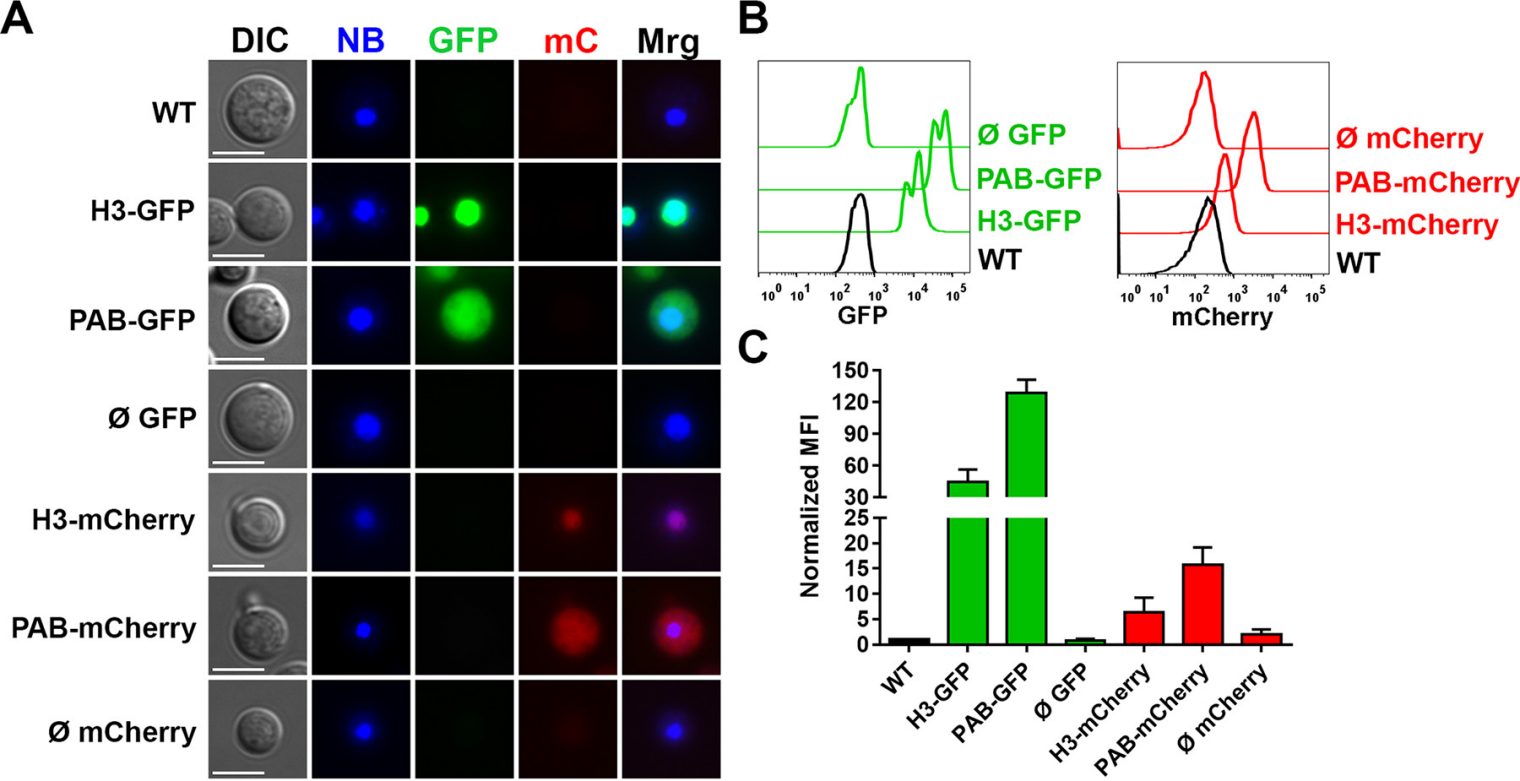
Engineered fluorescent strains exhibit a nuclear or cytoplasmic fluorescence distribution. (A) GFP or mCherry (mC) transformant strains were stained with the NucBlue (NB) nucleus probe and observed under a fluorescence microscope for assessment of the intracellular distribution of fluorophores (BF, bright field; Mrg, merge) (bars = 5 μm). (B and C) Fluorescence intensity histograms (B) and median fluorescence intensities (MFI) (C) of the transformant strains (normalized to the values for the WT) evaluated by flow cytometry. The results are presented as the means ± SD of data obtained from three independent experiments. Representative fluorescence micrographs were taken using automatic exposure, and histograms are adjusted to highlight the subcellular localization of the fluorescent fusion proteins, whereas the relative quantification of fluorescence signals was done by flow cytometry.

We observed that both H3-GFP and H3-mCherry transformants emitted green and red fluorescence, respectively, which colocalized with the nuclear marker NucBlue, indicating the successful localization of the fluorophores to this compartment (Fig. 1A). In contrast, PAB-GFP and PAB-mCherry transformants demonstrated a diffuse pattern of cytoplasmic staining limited by the plasma membrane.

Using flow cytometry, we detected a strong green fluorescence emission in H3-GFP and PAB-GFP transformants, with median fluorescence intensity (MFI) increases of approximately 45- and 130-fold (44.4- ± 23.8-fold and 128.5- ± 25.3-fold, respectively [means ± standard deviations {SD}]) in comparison to the WT strain, respectively (Fig. 1B and C). The H3-mCherry and PAB-mCherry transformants reached lower MFIs, approximately 6- and 16-fold (6.3- ± 5.1-fold and 15.7- ± 7.0-fold, respectively) higher than that of the wild type, respectively (Fig. 1B and C).

### The fluorescent strains exhibit no alterations in the stress response compared to the parental strain.

In both the environment and mammalian hosts, C. neoformans encounters diverse stress conditions, reacting to these conditions with adaptive responses and the expression of virulence-associated phenotypes. Within the mammalian host, two phenotypes largely determine a successful infection: growth at host physiological temperature (37°C) and resistance to leukocyte microbicide mechanisms, including reactive oxygen species (ROS) (38). Thus, we first evaluated these features by plating the fluorescent and WT strains at 30°C or 37°C onto yeast extract-peptone-dextrose (YPD) medium (Fig. S6A) and YPD medium supplemented with the oxidative stressors menadione (a superoxide anion donor) and H_2_O_2_ (Fig. S5B). All transformants exhibited phenotypes similar to that of the WT strain in response to thermal and oxidative stresses (Fig. S6A and B, respectively).

We also evaluated susceptibility to compounds that alter cell wall assembly or affect membrane permeability. We plated serially diluted yeast cells of the fluorescent strains and the WT on YPD medium supplemented with SDS, a detergent that destabilizes the cellular plasma membrane; Congo red, which prevents the biosynthesis of structural cell wall polysaccharides (39); and sodium chloride, which increases the osmolarity of the medium (Fig. S6C and D). None of the fluorescent strains had altered susceptibility after exposure to these stress conditions compared to the WT strain. To assess the growth kinetics of the fluorescent strains at 30°C in liquid YPD medium, we spectrophotometrically quantified growth for 96 h (Fig. S7). All strains had equivalent growth curves, reaching stationary phase without significant differences in growth rates or doubling times, except for PAB-mCherry, which showed a slight growth delay in comparison to the WT, reaching a lower plateau after 96 h of incubation.

### The fluorescent strains preserve the production of mating structures.

**FIG 2:**
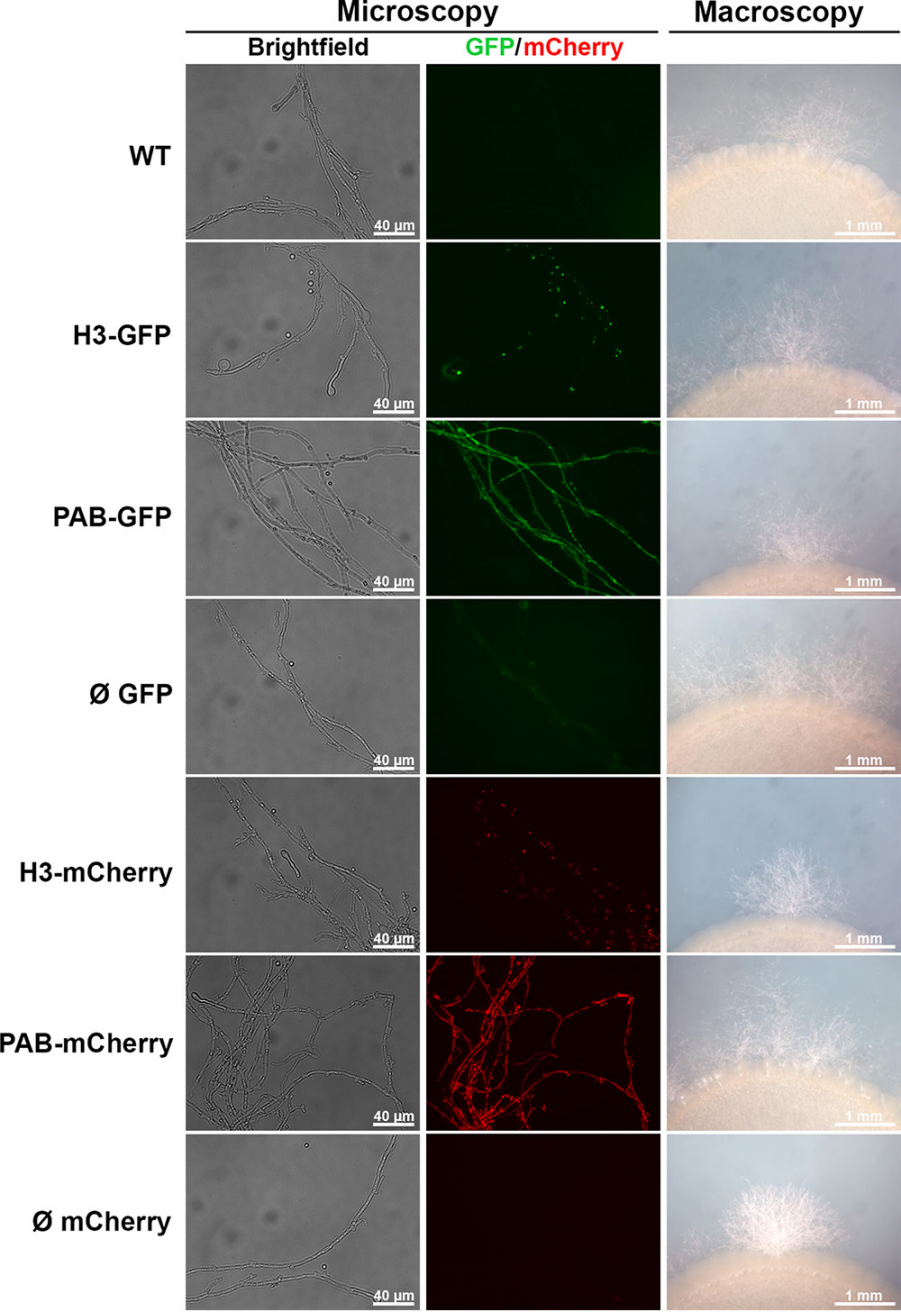
The fluorescent strains preserved the production of mating filaments. The transformant strains were mixed with an equal amount (10 μL of a suspension of 1 × 10^7^ cells/mL) of KN99 cells (MATα) and cocultured on agar filament plates for 7 days at room temperature. Hyphal fragments were assessed by fluorescence photomicroscopy at a ×200 magnification using automatic exposure, and histograms are adjusted to highlight the fluorescent fusion proteins. The edges of mating colonies were photographed with a stereomicroscope under a ×2.5 magnification.

In order to verify the impact of the genomic integration of the GFP and mCherry cassettes on the C. neoformans bipolar mating life cycle, the fluorescent strains, which are MATα, were cocultured with the KN99*a* strain (MAT**a**) on filament agar, and we evaluated the ability of the strains to form dikaryotic mating hyphae (Fig. 2). In all spots of cocultivation, we detected fluorescent mating hyphae in equivalent amounts. In addition, intact clamp connections were visualized along the filaments, which are important features of mating hyphae in many basidiomycete fungi. Importantly, the subcellular localization of the fluorescent fusion proteins within the hyphal cells was similar to that in the yeast-like cells: H3 fusion proteins localized to the nuclei, and PAB fusion proteins localized to the cytoplasm (Fig. 2). Finally, we also evaluated the ability of the fluorescent strains to undergo meiosis on MS (Murashige-Skoog) agar. After microscopic inspection, we detected the production of basidia and basidiospore chains, consistent with intact and complete sexual development (Fig. S8).

### Expression of virulence-associated phenotypes.

**FIG 3:**
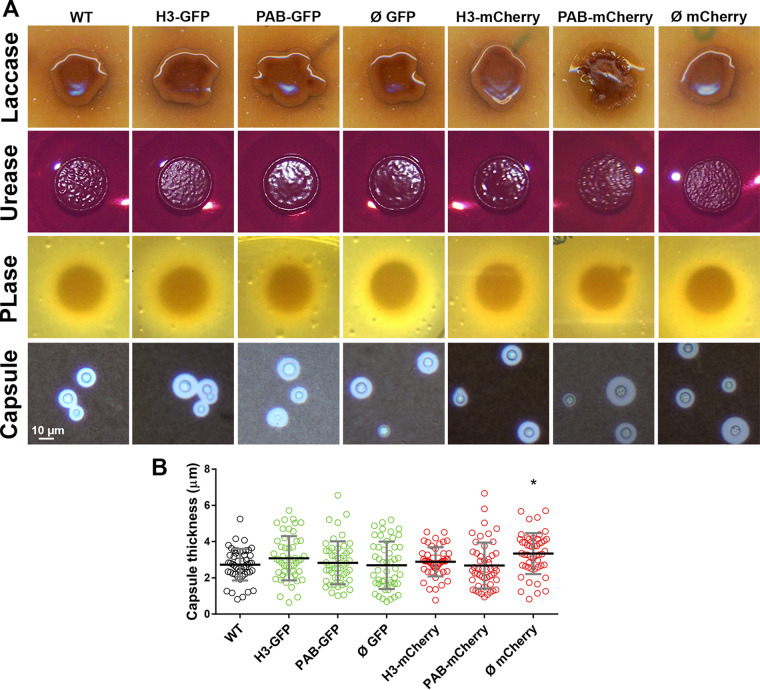
Expression of virulence-associated phenotypes. (A) Transformant strains were evaluated for laccase-dependent melanization (Niger seed agar), urease (Christiansen’s urea agar) and phospholipase (PLase) (egg yolk agar) activities, and capsule formation (growth in liquid minimal medium and Indian ink preparation). (B) Measurement of capsule thickness was performed based on the formation of the ink exclusion zone observed under a light microscope. Black bars represent the means ± SD of data pooled from two experiments. *, *P* < 0.05 (compared to the WT).

The Cryptococcus polysaccharide capsule protects yeast cells from dehydration and has several roles in the interactions with mammalian and invertebrate hosts (40). After capsule induction, we observed by India ink counterstaining that all of the fluorescent strains showed similar thicknesses of the capsule, except for the Ø mCherry strain, which exhibited a modest capsule enlargement compared to the WT strain (Fig. 3A and B). Other well-characterized virulence determinants in C. neoformans were also evaluated in the fluorescent strains. No difference among the strains was noted for melanin production on Niger seed agar plates: all strains produced the dark pigment resulting from the oxidation of phenolic compounds by laccase (41). The production and activity of the secreted enzymes phospholipase and urease were likewise similar to those of the WT (Fig. 3A). Our results indicate that the fluorescent strains preserved the WT expression levels of many virulence-associated phenotypes.

### The fluorescent strains are virulent in a wax moth model and survive after phagocytosis by activated murine macrophages.

**FIG 4:**
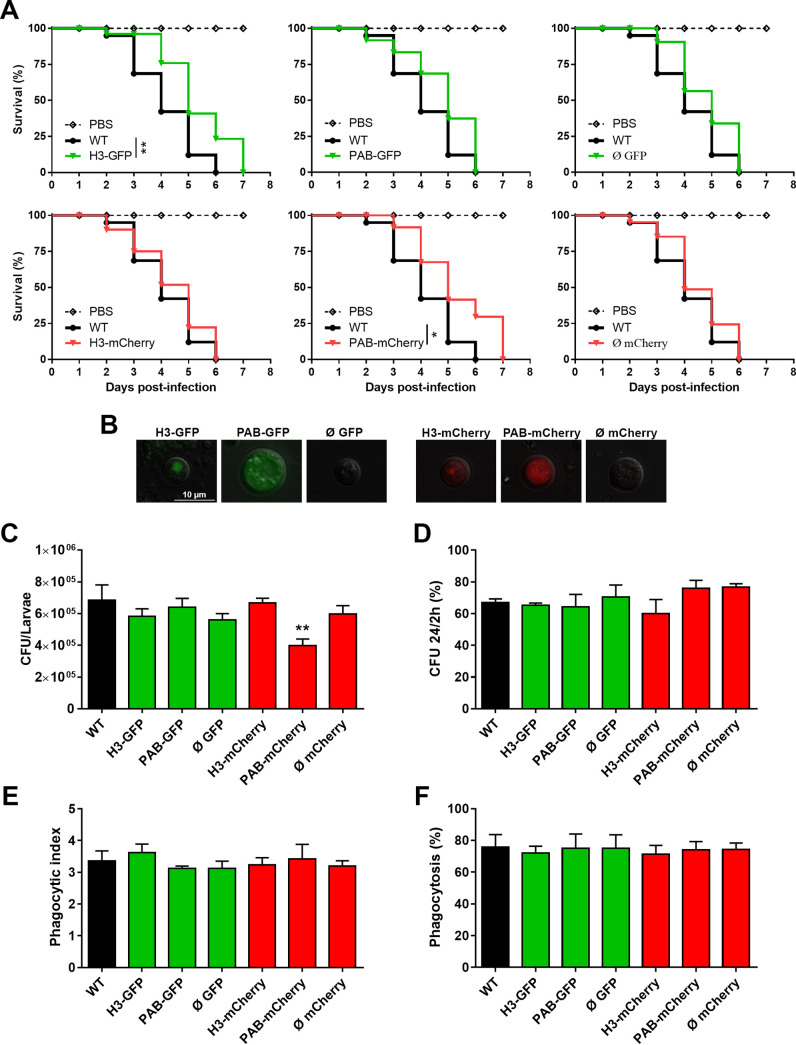
The fluorescent strains are virulent in a wax moth model and survive after phagocytosis by activated murine macrophages. (A) G. mellonella caterpillars (*n* = 14 to 17) were inoculated with either PBS alone (inoculation trauma control) or 5 × 10^4^ yeast cells from each transformant strain. (B) After 5 days of infection, hemolymph was recovered from individual larvae for analysis of yeast by fluorescence microscopy. (C) Fungal burdens in G. mellonella caterpillars (*n* = 5) at the LT_50_ upon infection with the transformant strains. (D) Primary murine macrophages were activated for 3 h with LPS (1 μg/mL) and IFN-γ (20 ng/mL) and inoculated (MOI of 2) with the previously opsonized transformant strains. After 2 and 24 h of interaction, the cell monolayer was washed and lysed, and the resulting yeast suspension was diluted and seeded onto SDA plates for CFU counting. The percentage of fungal survival was calculated based on the 24-h/2-h CFU ratio multiplied by 100. The results are representative of data from two independent experiments. (E and F) For phagocytosis analysis, nonactivated primary murine macrophages were infected at an MOI of 5. After 2 h of interaction, the cell monolayer was stained with a panoptic stain for analysis of the phagocytic index (yeast cells/macrophages) and the percentage of phagocytosis (percentage of macrophages containing yeast) by light microscopy. The results are presented as the means ± SD and are representative of data from two independent experiments. *, *P* < 0.05; **, *P* < 0.01 (compared to the WT).

The fluorescent strains were evaluated for virulence in an invertebrate animal model of fungal virulence (Fig. 4A). After the inoculation of G. mellonella larvae with each C. neoformans strain, host mortality was monitored daily. All larvae in most of the groups died on the 6th day after inoculation, but the groups inoculated with the H3-GFP and PAB-mCherry strains exhibited slightly prolonged survival (7 days). On the 5th day of infection, we recovered hemolymph from infected larvae that succumbed that day, and the visualized yeast cells retained the ability to emit red or green fluorescence throughout the course of the infection (Fig. 4B). In addition, we determined the fungal burden at the average time to lethality for 50% of the insects (LT_50_) (3.82 ± 0.42 days) (Fig. 4C). Only the PAB-mCherry group showed a decreased fungal load compared to that of the parental strain, in agreement with the survival curve data (Fig. 4A), thus confirming that this specific mutant is hypovirulent in the G. mellonella model.

We also conducted cocultures of activated primary bone marrow-derived murine macrophages and antibody-opsonized C. neoformans yeast cells. The fungal cell survival rate was analyzed by quantitative culture after 2 and 24 h of interaction. There was no statistically significant difference in the fungal loads among the experimental groups (Fig. 4D). In addition, we assessed the phagocytic index and percent phagocytosis of the murine macrophages by light microscopy. There was no difference in these measures of engulfment between the fluorescent strains and the WT (Fig. 4E and F). The phagocytic index was about 3 yeast cells per macrophage, while the phagocytosis rate for all strains was approximately 80%. Based on the results presented here, our data support that the fluorescent C. neoformans strains will likely display similar virulence-related activities both *in vitro* and in direct host interaction studies.

### Use of the PAB-GFP strain as a tool to improve knowledge on C. neoformans-host interaction dynamics.

To explore the applicability of the fluorescent strain to host-pathogen interaction analyses, murine macrophages were cocultured with the PAB-GFP strain. This strain was chosen due to its highest fluorescence emission among all of the obtained strains. Our approach was based on fluorescence detection by microscopy and flow cytometry using fluorescein isothiocyanate (FITC)-labeled WT C. neoformans as a control.

**FIG 5:**
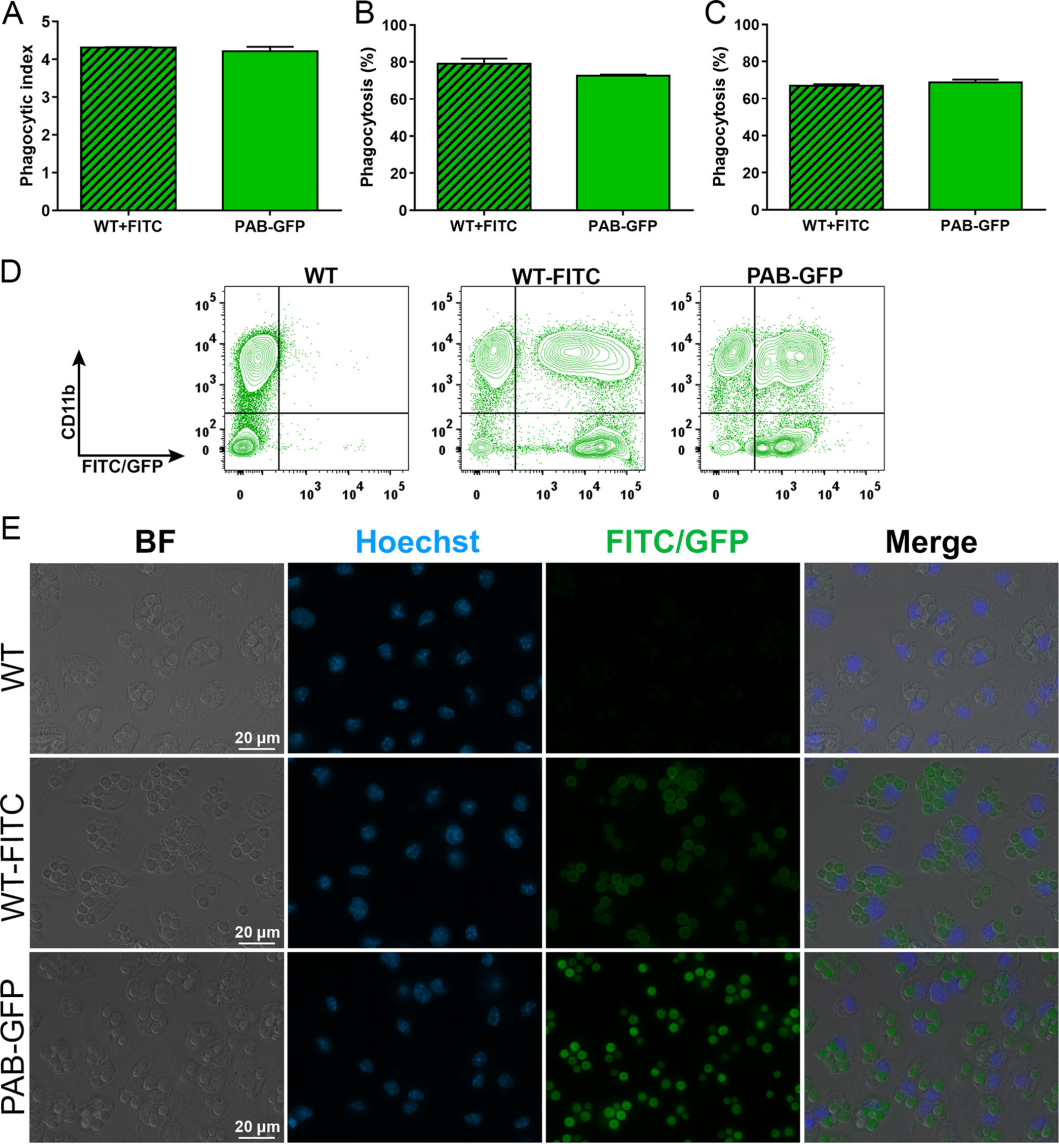
The transformant strains show no difference from the parental strain in phagocytosis by murine macrophages by fluorescence-based analysis. (A and B) Primary murine macrophages were cocultured at an MOI of 5 with the WT, FITC-labeled WT, or PAB-GFP strain previously opsonized with mAb 18B7. After 2 h of interaction, the phagocytic index (yeast cells/macrophages) and percentage of phagocytosis (percentage of macrophages containing yeast) were evaluated by fluorescence microscopy. (C) For analysis of the percentage of phagocytosis by flow cytometry, the cell monolayer was washed, detached, and stained with anti-CD11b antibody conjugated to APC for the identification of macrophages. (D) Flow cytometry dot plots of CD11b-APC versus FITC/GFP. (E) Fluorescence micrographs of the interaction between macrophages and yeast cells under a magnification of ×400. The results are presented as the means ± SD and are representative of data from two independent experiments.

Fluorescence microscopy analyses of the macrophage phagocytic index and the percentage of these cells engaged in phagocytosis did not show any statistical difference between the PAB-GFP and WT strains (Fig. 5A and B), which is in agreement with the results obtained by the use of light microscopy (Fig. 4D and E). Phagocytosis was also assessed by flow cytometry (Fig. 5C) by the use of a protocol that combines the labeling of macrophage-1 antigen (CD11b) using a monoclonal antibody (mAb) conjugated to the far-red fluorophore allophycocyanin (APC) and GFP/FITC labeling of C. neoformans. We noted two well-delimited populations of macrophages, either containing (CD11b positive [CD11b^+^] FITC/GFP^+^) or not containing (CD11b^+^ FITC/GFP negative [FITC/GFP^−^]) internalized yeast cells (Fig. 5D). Extracellular yeast cells (CD11b^−^ FITC/GFP^+^) were also detected. Once again, we noted no difference between the rates of phagocytosis of the PAB-GFP and WT strains (Fig. 5C). Our results support the use of C. neoformans PAB-GFP coupled with fluorescence microscopy and flow cytometry as a useful and reliable technique for the quantitative analysis of C. neoformans-host interactions.

### C. neoformans PAB-GFP cultured under nutrient starvation and hypoxia exhibits the viable but nonculturable cell phenotype and stable GFP expression.

**FIG 6:**
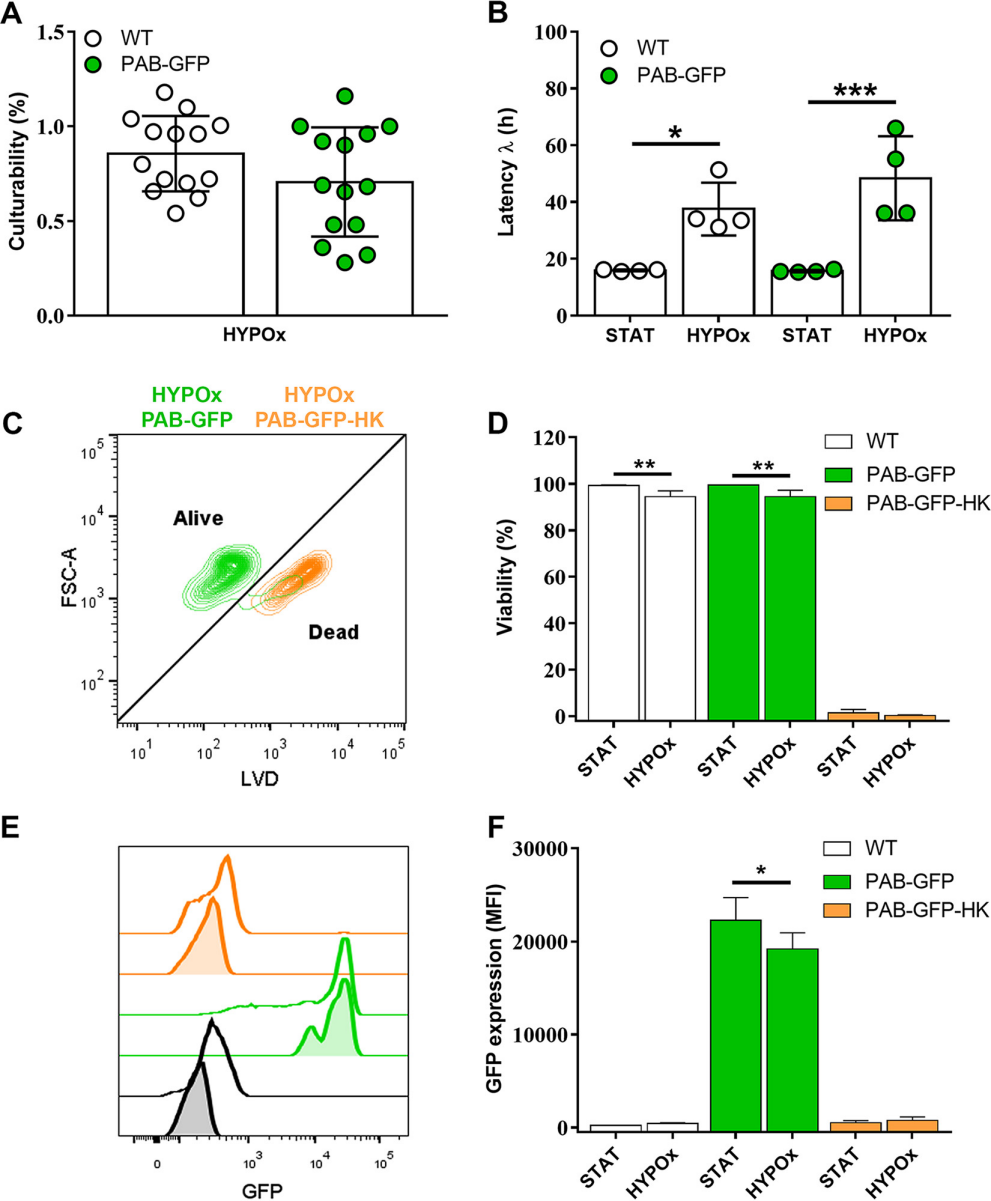
C. neoformans PAB-GFP grown under nutrient starvation and hypoxia exhibits the viable but nonculturable cell phenotype and stable GFP expression by flow cytometry. (A and B) WT or PAB-GFP yeast cells in stationary phase (STAT) or incubated for 7 days under hypoxia (HYPOx) were inoculated onto SDA plates for CFU counting, which was used to determine the percentage of culturable cells (A), or inoculated into fresh liquid medium for analysis of growth latency (B). (C and D) Cells were labeled with LIVE/DEAD violet dye (LVD) for cell viability analysis by flow cytometry. (E and F) GFP expression in viable or heat-killed (HK) cells was evaluated. In panel E, filled histograms denote cultures of STAT yeast cells, while empty histograms represent HYPOx cells. FSC-A, forward scatter area. The results are presented as the means ± SD from at least two independent experiments. *, *P* < 0.05; **, *P* < 0.01; ***, *P* < 0.001.

Recently, the induction of viable but nonculturable (VBNC) C. neoformans yeast cells has been characterized *in vitro* (21). Due to the relevance of this dormant phenotype for the pathophysiology of cryptococcosis, we used the laboratory protocol reported previously by Hommel et al. to assess the ability of the PAB-GFP strain to enter dormancy by first incubating cells to stationary phase (STAT) and nutrient depletion, followed by the prolonged exposure of the culture to hypoxia (HYPOx) (21). At the end of the incubation period, HYPOx or control yeast cells (STAT) were inoculated into fresh solid or liquid medium to assess growth capacity (culturability, by CFU counting) and latency (i.e., lag or λ phase), respectively. True VBNC cells must exhibit an impact on both parameters (21). In our experiments, we observed that both WT and PAB-GFP yeast cells experienced a drastic reduction in culturability to levels below 1%, in agreement with previously reported observations (21) (Fig. 6A). We monitored growth in liquid medium and determined the length of the lag phase, observing a significant delay in the growth of HYPOx cells (Fig. 6B). Both PAB-GFP and WT cells remained in the latency phase approximately 2 to 3 times longer (37.5 h ± 9.3 h and 48.3 h ± 14.8 h, respectively) than cells that were not subjected to hypoxia, which required a shorter period of approximately 16 h (15.9 h ± 0.3 h and 15.6 h ± 0.5 h, respectively) to reestablish vigorous growth.

In parallel, we also verified the viability of yeast cells from stationary phase or those collected under hypoxia conditions using an amine-reactive violet fluorescent dye (LIVE/DEAD violet dye [LVD]) coupled with flow cytometry (Fig. 6C and D). As a control, we used heat-inactivated (heat-killed [HK]) yeast cells. Our data reveal that WT and PAB-GFP HYPOx yeast cells have high and equivalent levels of viability (94.5% ± 2.5% and 94.5% ± 2.7%, respectively), with a slight reduction in comparison to their respective STAT conditions (99.3% ± 0.3% and 99.5% ± 0.1%, respectively).

**FIG 7:**
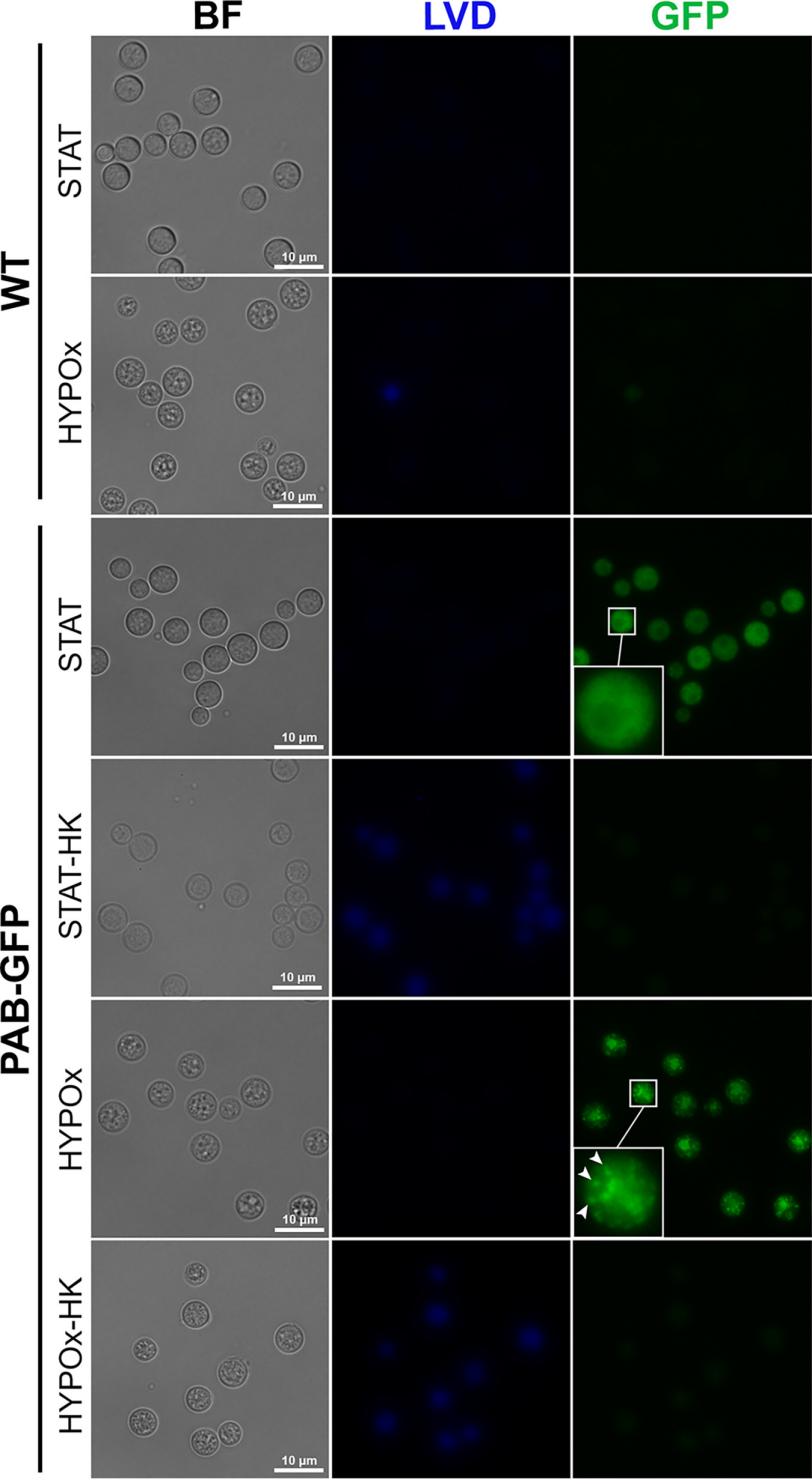
C. neoformans PAB-GFP VBNC cells display GFP expression and a specific cell distribution pattern by fluorescence microscopy. WT or PAB-GFP yeast cells in stationary phase (STAT) or in a hypoxic atmosphere (HYPOx) were heat killed (HK) or not, subjected to LIVE/DEAD Violet Dye (LVD) staining, and analyzed for cell viability and GFP expression by fluorescence microscopy. Dead cells appear blue. The arrowheads indicate stress granules. The results are representative of data from two independent experiments.

Finally, we used flow cytometry to determine if GFP expression and its intensity would be affected by the conditions to which the cells were subjected. Interestingly, PAB-GFP yeast cells showed robust fluorescence emission (MFI of 19,188.8 arbitrary units [AU] ± 1,739.8 AU), comparable to the fluorescence emitted by STAT cells (22,289.5 ± 2,427.2 AU) (Fig. 6E and F). Both STAT and HYPOx PAB-GFP HK yeast cells displayed intense staining with the viability dye (indicative of cell death) and, simultaneously, the almost total elimination of the green fluorescence signal (519.8 ± 222.6 AU and 756.0 ± 378.2 AU, respectively), presumably due to the thermal degradation of GFP. The same fluorescence emission patterns for the viability marker or GFP were confirmed by fluorescence microscopy (Fig. 7). To our knowledge, this is the first work that characterizes a fluorescently engineered strain of C. neoformans that is able to enter a dormant state and remain traceable in the long term.

### C. neoformans PAB-GFP VBNC cells show a specific fluorescence distribution pattern.

Although found predominantly in the cytosol, Saccharomyces cerevisiae Pab1 is regularly imported to the nucleus to engage in the biogenesis of mRNA and its transport to the cytosol, thus controlling protein translation. Under certain stressful conditions, translation is committed, simultaneously with the cell redistribution of Pab1 (and Pab1-bound transcripts), in structures known as stress granules (SGs) (36).

**FIG 8:**
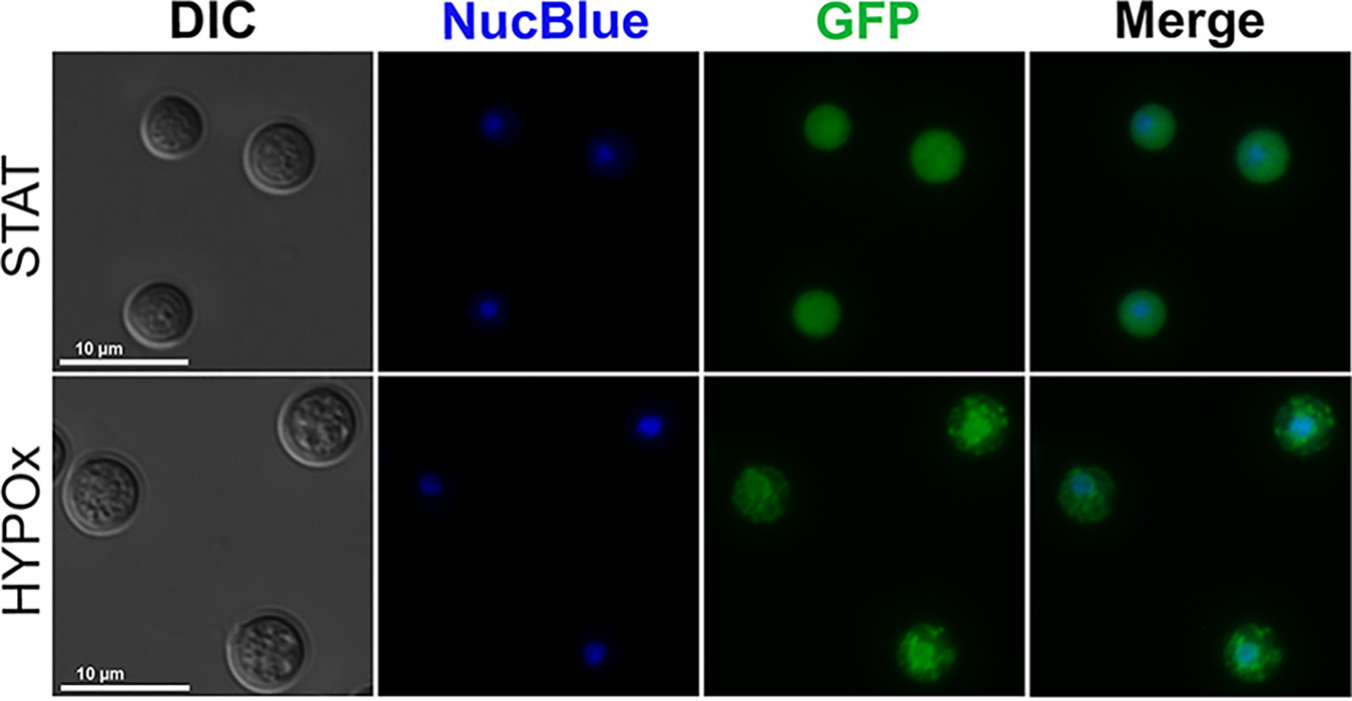
C. neoformans PAB-GFP VBNC cells exhibit accumulation of Pab1 in stress granules and the nucleus. WT or PAB-GFP yeast cells were grown until stationary phase (STAT) and then incubated for 7 days in a hypoxic atmosphere (HYPOx). Nuclei (blue) were stained with NucBlue. The results are representative of data from two independent experiments.

During fluorescence microscopy analysis of the fluorescent strain PAB-GFP in the VBNC state, we observed a distinct intracellular distribution pattern of GFP-tagged Pab1, not diffusely within the cytoplasm, as we detected on STAT cells, but consisting of cytoplasmic structures consistent with SGs (Fig. 7). We also noted an accumulation of fluorescence signals in the nucleus (Fig. 8). To confirm this observation, we stained the cells with the NucBlue DNA probe, and we were able to detect the colocation of the nuclear compartment with a subset of the Pab-GFP punctate signals during the VBNC state (Fig. 8). Altogether, our data indicate that Pab1 mediates a different mechanism of posttranscriptional regulation, the formation of SGs and the nuclear retention of Pab1, in C. neoformans VBNC cells in response to the physiological stresses involved in entry into dormancy.

## DISCUSSION

Originally identified in the jellyfish Aequorea victoria (42), GFP, as well as its fluorescent counterparts, has been widely expressed in a broad range of engineered microorganisms, including fungi, to study their biology and interactions with hosts (43). The use of fluorescent strains, whether containing GFP or mCherry, is not unprecedented in the research on Cryptococcus-host interactions. Voelz et al. (44) developed GFP-expressing mutants of reference strains of C. neoformans and Cryptococcus gattii that improved automated analyses of intracellular proliferation, phagocytosis, and nonlytic exocytosis. Afterwards, Upadhya and collaborators (45) engineered a red-emitting derivative of the H99 congenic strain KN99 using mCherry, which allowed fluorescence-based *ex vivo* determinations of intracellular and extracellular lung fungal burdens during experimental murine infection. Recently, Spencer and colleagues (46) expanded the current red-green color palette of engineered fluorescent C. neoformans strains. Despite not being as fluorescently robust as the above-mentioned transformant strains, our PAB-GFP transformant also showed low background fluorescence, was successfully used to aid in analyses of C. neoformans-host interactions, and, importantly, allowed a more detailed exploration of interesting features of the biology of C. neoformans, including the recently described viable but nonculturable cell phenotype.

The advantages of transgenic labeling over conventional cell labeling (fluorescently labeled antibodies and probes) are many and have been corroborated in this work (43, 47). These include highly stable fluorescence emission, which does not decrease upon cell division, allowing long-term analysis, a major limitation of conventional labeling fluorophores (e.g., FITC, 5-chloromethyl fluorescein diacetate [CMFDA], Uvitex-2B, and calcofluor). In fact, our transformant strains showed fluorescence emissions even after 5 days of *in vivo* infection using the *Galleria* model.

Another advantage of GFP labeling is the possibility of correlations between cell death and the loss of the GFP signal that follows, a strategy often explored in models of GFP-expressing bacteria. This approach allows viable-cell enumeration rather than by quantitative culture (48–50). This feature is especially useful when studying phenotypes such as the VBNC state, in which CFU may not perfectly reflect the cellular state (50). All dead yeast cells resulting from heat treatment or obtained from the protocol to induce VBNC exhibited a loss of GFP signal (Fig. 6). Therefore, the PAB-GFP strain could be useful for the automated quantification of fungal loads instead of CFU counting, which is time-consuming, labor-intensive, and unable to detect VBNC cells.

On the other hand, a concern for all genetically engineered strains is the risk of unintended mutations introduced during transformation. Strategies such as the insertion of constructs within intergenic genomic regions, such as the “safe haven” (SH) region, help to minimize these side effects (37). However, they do not exclude unexpected impacts on virulence, as was observed previously for an mCherry mutant containing the targeted construct for the safe haven 2 (SH2) locus (45). It is important to highlight that the insertion of exogenous DNA into the SH locus is not simple to achieve. In our experience, of the 32 mutants analyzed, only 4 (12.5%) had the fluorescence cassette present in the SH locus. Of the six mutants previously selected for this study, only Ø GFP presented the cassette located at this site of the genome (see Fig. S5 in the supplemental material). Therefore, evaluations of a series of established virulence attributes and growth in response to stress, such as those conducted in the present study, are prudent after genetic transformations performed in C. neoformans. Our strains were similar to the WT in these plate-based tests, even though we had no control over the locus integration of the constructs or the number of copies inserted into the genome. PAB-mCherry, however, showed hypovirulence in the invertebrate animal model G. mellonella, which could be related to its decreased growth compared to that of the WT strain. Moreover, we demonstrated that our transformants are virulent and exhibit stable fluorescence in *in vivo* infections in the invertebrate animal model of G. mellonella-fungus interactions. Most importantly, the transformant and parental strains also experienced similar rates of killing after phagocytosis by activated murine macrophages, which presents a high correlation with the data from a mouse survival assay (51).

In addition to their use in the identification and tracking of cells in interaction assays, fluorescent proteins are valuable in the construction of fungal reporter strains and for monitoring the expression of genes of interest as well as the cytolocalization of their protein products. Furthermore, this approach allows the description of relevant genes for fungal biological processes and interactions with host cells (43). A myriad of reporter strains has been engineered in C. neoformans since del Poeta and collaborators (24) fused GFP to the alpha-mating-type MFα1 promoter, with the purpose of studying the expression of this gene in the course of infection. Among the fluorescent reporters that followed, RAS-mCherry was applied to the zebrafish model, demonstrating the potential for chimera transformants, as we did here using GFP, to assist in C. neoformans-host interaction studies (52, 53). In our study, we chose the histone H3 promoter to achieve the strong constitutive expression of GFP and mCherry.

Here, we demonstrated a set of transformants containing fluorophores of low spectral overlap (GPF or mCherry) (54), directed to two different cell compartments (cytoplasm or nucleus), and capable of maintaining the pattern of fluorescence emission in different C. neoformans morphotypes, including hyphae. Furthermore, for the first time, fluorescent transformants of C. neoformans were used in an assay to generate VBNC cells, using a recently described protocol based on the combination of nutrient- and oxygen-limiting conditions (21). This technique is an important tool in the study of the biology of these cells and latent cryptococcal infection, especially when considering that obtaining dormant C. neoformans cells was previously dependent on using experimentally infected animals and cell sorting, with an extremely low yield, making the procedure unfeasible for many applications (20).

We demonstrated that cultures of PAB-GFP submitted to the *in vitro* induction of dormancy successfully reproduced defining criteria for viable but nonculturable cells, such as growth latency and low culturability (21, 55). Moreover, VBNC yeast cells maintained GFP emission even during low metabolic activity and after prolonged incubation, especially in a hypoxic atmosphere. GFP chromophore maturation involves intramolecular reactions in the presence of oxygen, so low oxygen concentrations can prevent the formation of the fluorescent chromophore (56–58). However, some studies, like ours, have overcome known limitations of the use of GFP under hypoxic conditions (58, 59). Hansen and collaborators (60) observed that biofilms of Streptococcus gordonii exhibited an extremely low minimum oxygen requirement of 0.1 ppm for GFP maturation. In addition, even after the complete loss of the GFP signal when cells were grown under lower oxygen levels, cell fluorescence was fully recovered in minutes following sample reoxygenation. Thus, it is possible that the oxygenation levels reached with the protocol used here were not low enough to impact GFP fluorescence. Alternatively, it is plausible that the fluorophore signal was recovered immediately after the yeast was reexposed to normoxia.

An interesting feature that we found in dormant PAB-GFP yeast cells was the cytoplasmic distribution pattern of Pab1, characteristic of ribonucleoprotein granules. The formation of such nonmembranous granules composed of RNA and proteins is an adaptive response of eukaryotes to diverse conditions of cell stress, occurring together with a decline in translation processes (61, 62). Ribonucleoprotein granules include processing bodies and stress granules (SGs). Whereas the former has historically been described as a transcript degradation site, SGs are believed to regulate mRNA storage, protection from decay, and translation silencing and reinitiation (61–64). Moreover, SGs contribute to cell survival by inhibiting apoptosis (65, 66). Pab1 is considered a physiological stress sensor and a classical SG marker, often in its GFP-fused form (36, 62, 67–69), as is the case in this study. In S. cerevisiae, a *PAB1* gene deletion results in a lower frequency of SGs (70), whereas its overexpression promotes larger and longer-lasting SGs, inducing stress-tolerant phenotypes (68). In C. neoformans, until the present work, there were descriptions of the presence of Pab1 in SGs only in response to thermal and osmotic stresses (71), although in other yeasts, Pab1 is involved in responses to stresses such as the depletion of carbon sources (glucose) and inhibition of the mitochondrial respiratory chain (67, 72).

Here, we demonstrate that C. neoformans VBNC cells induced by both nutrient and oxygen depletion exhibit the formation of stress granules. This finding may suggests a possible role of stress granule assembly in dormancy maintenance and the survival of VBNC cells. Phase separation of Pab1 and other SG-associated proteins, a process different from but related to SG formation, also confers fitness under adverse conditions in other organisms (69, 73, 74). In addition, the resulting solid-like state of the cytoplasm is associated with entry into dormancy (75) and can be reversed, which coincides with the continuation of the cell cycle and cell reactivation from quiescence (64, 76). Likewise, monitoring the formation and dissolution of SGs by fluorescence-based techniques could assist in understanding the dynamic physiology of entry into and exit from dormancy as well as identifying molecules that have a role in these processes.

In C. neoformans, supplementation of VBNC yeast cells with pantothenic acid, a precursor for acetyl-CoA, increases the frequency and speed of reactivation (21). Although the cytoplasmic structural changes linked to this reactivation have not been investigated, acetyl-CoA levels have been shown to regulate SG formation in S. cerevisiae subjected to glucose deprivation (77). Tests that evaluate the ability to dissolve PAB-GFP stress granules by cell incubation with potential reactivation stimuli may be useful to clarify both the association between stress granules and dormancy as well as the description of dormancy inducers and reactivation factors.

Interestingly, we observed clusters of PAB-GFP not only within the cell cytosol but also inside the nucleus of C. neoformans cells. Although mostly found diffusely throughout the cytosol, Pab1 is regularly imported by the nucleus to act on mRNA biogenesis and the export of mRNA for translation (36, 78). Interestingly, the restriction and accumulation of this protein in the nucleus have already been described under different stress conditions in human cells and exert an impact on protein synthesis (79–81). During dormancy induction, C. neoformans yeast cells undergo a reduction in the diversity of proteins concomitantly with an increase in their concentration, suggesting that, although generally suppressed, transcription and translation processes are maintained in this cellular state (21). Recently, nuclear mRNA retention in parallel with the formation of cytoplasmic SGs has been described in human cells under stress induced with tubercidin (82). In that study, the authors attributed the accumulation at the nuclear site to transcripts formed during stress, while the reallocation to cytoplasmic granules was attributed to those produced prior to treatment with tubercidin. We believe that the same pattern may be occurring in our model.

Our results support the use of PAB-GFP in *in vitro* and *in vivo* infection models that explore the use of fluorescence-based techniques, especially as it is the first engineered fluorescent lineage of C. neoformans proven to be able to engage in dormancy and, mostly, remain traceable. Furthermore, this strain can be particularly useful for determining fungal loads in infections conducted with dormant inocula since the conventional CFU quantification methodology is not adequate due to viable but nonculturable cells.

In addition, our results indicate that Pab1 mediates different mechanisms of posttranscriptional regulation in dormant C. neoformans cells in response to the physiological stresses involved in entry into dormancy, which may be implicated in both the survival and phenotypic maintenance of these cells and the dynamics of the exit from dormancy. Our work also paves the way for the use of GFP (or other fluorescent proteins) as a reporter molecule to investigate the behavior, cytolocalization, and biological role of proteins of interest in the dormancy and reactivation of C. neoformans, processes of great relevance in the pathophysiology of cryptococcosis (6, 22, 83).

## MATERIALS AND METHODS

### Culture conditions for C. neoformans and Escherichia coli.

Wild type (WT) strain H99O of C. neoformans (var. *grubii*), kindly provided by Andrew Alspaugh from Duke University, and transformants were grown at 30°C under agitation at 120 to 150 rpm in YPD broth (1% yeast extract, 2% glucose, and 2% peptone) or solid (2% agar) medium. Escherichia coli was grown under agitation at 150 to 200 rpm in liquid LB medium (1% peptone, 0.5% NaCl, 1% yeast extract) and solid LB medium (1.5% agar) at pH 7.2.

### Construction of the fluorescence cassettes.

The C. neoformans *H3* (CNAG_06745) and *PAB* (CNAG_04441) sequences encoding histone H3 and poly(A) binding protein were obtained from FungiDB (https://fungidb.org/fungidb/) for primer design and amplification by PCR. All primer sequences used in this work are described in Table S1 in the supplemental material. The amplification reaction was performed using a final volume of 25 μL containing 50 ng of the genomic DNA (gDNA) template (from H99), 1× OneTaq polymerase master mix (New England BioLabs [NEB]), and 0.4 μM each primer pairs LF498/LF499 and LF502/LF503 for the *H3* and *PAB* genes, respectively, and their terminator sequences. The following temperature cycles were used: an initial denaturation step at 94°C for 30 s, 35 cycles of denaturation at 94°C for 30 s each, annealing at 55°C for 30 s, an extension step at 68°C for 3 min, and a final extension step at 68°C for 10 min. The final products of 856 bp (*H3*) and 2,941 bp (*PAB*) with BamHI restriction sites at their ends were purified from 1% agarose gels using a QIAquick gel extraction kit (Qiagen).

Cloning of the fragments was performed using pGEMT Easy (Promega), according to the manufacturer’s instructions. The ligation mixtures were transformed into E. coli DH5α by heat shock at 42°C for 90 s. After the selection of the colonies with the cloned fragments, 12 μg of each plasmid was digested with BamHI-HF (NEB). The plasmids pCN50 and pCN51, which possess the eGFP and mCherry genes under the control of the *H3* promoter (P*_H3_*) (84), respectively, were digested with BamHI-HF (NEB) and then dephosphorylated with Antarctic phosphatase reaction buffer (NEB). The dephosphorylated plasmids (pCN50 and pCN51) and the purified 0.85-kb *H3*-T*_H3_* and 2.94-kb *PAB*-T*_PAB_* fragments were ligated using T4 DNA ligase (NEB), according to the manufacturer’s instructions. After the ligations, E. coli heat shock transformations were performed, and plasmids were extracted from the colonies.

To check the orientation of the genes (*H3* or *PAB*), digestions with specific restriction enzymes were performed. pCN50/GFP-*H3* and pCN50/GFP-*PAB* were digested with KpnI (NEB), pCN51/mCHERRY-*H3* was digested with HindIII (NEB), and pCN51/mCHERRY-*PAB* was digested with BglII. All expected patterns were confirmed by electrophoresis on 1% agarose gels and visualized under UV light with ethidium bromide (0.5 μg/mL). For all constructs, *H3* or *PAB* was cloned in frame at the 3′ end of the GFP or mCherry gene.

The cassettes P*_H3_*-GFP-*H3-*T*_H3_* (2.3 kb), P*_H3_*-mCherry-*H3-*T*_H3_* (2.3 kb), P*_H3_*-GFP-*PAB-*T*_PAB_* (4.4 kb), P*_H3_*-GFP (1.5 kb), and P*_H3_*-mCherry (1.5 kb) were amplified using *Taq* LongAmp master mix (NEB) and 0.2 μM each primer (LF512/LF513) with the following cycling conditions: an initial denaturation step at 94°C for 1 min, 35 denaturation cycles at 94°C for 30 s each, an annealing step at 59°C for 1 min, an extension step at 65°C for 4 min, and a final extension step at 64°C for 10 min. P*_H3_*-mCherry-*PAB-*T*_PAB_* was obtained by digestion with HindIII-HF and SpeI (NEB). Plasmid pSDMA57 (Neo^r^) (37) was digested with ClaI and SacII (NEB) for further recombination of the fragments P*_H3_*-GFP-*H3-*T*_H3_* (2.3 kb), P*_H3_*-GFP-*PAB-*T*_PAB_* (4.4 kb), and P*_H3_*-GFP (1.5 kb) using an In-Fusion PCR cloning kit (Clontech). Plasmid pSDMA58 (hygromycin B resistance) (37) was digested with ClaI and SpeI (NEB) for the P*_H3_*-mCherry-*H3-*T*_H3_* (2.3-kb) and P*_H3_*-mCherry (1.5-kb) insertions. P*_H3_*-mCherry-*PAB-*T*_PAB_* (4.2 kb) was released after pCN51/mCHERRY-*PAB* digestion with HindIII and SpeI (NEB) and cloned using T4 DNA ligase (NEB) into pSDMA58 previously digested with the same restriction enzymes. The recombination and ligation reaction mixtures were heat shocked in E. coli cells. The obtained plasmids containing the fluorescence genes fused to *H3* or *PAB* were confirmed by restriction enzyme reactions and linearized with AscI (NEB) prior to transformation into C. neoformans. Strains transformed with the pSDMA57 or pSDMA58 plasmid carrying GFP or mCherry not fused to any gene were used as negative controls (Ø GFP and Ø mCherry, respectively). See Fig. S1 and S2 in the supplemental material for the complete flowchart depicting all of the steps for the construction of the cassettes.

### Biolistic transformation of C. neoformans.

C. neoformans H99 was cultured in 50 mL of liquid YPD medium for 24 h at 120 rpm at 30°C. The culture was centrifuged at 4,000 rpm for 5 min, and the pelleted cells were washed once and resuspended in 5 mL of a sterile saline solution (0.9% NaCl). One milliliter of the cell suspension was spread onto plates containing YPD medium plus 1 M sorbitol and allowed to dry in a sterile laminar flow hood at room temperature for 1 h. Treatment with tungsten particles to coat DNA was done as described previously (85). Briefly, each treatment consisted of 50 μL of a sterile tungsten microcarrier (60 mg/mL) (0.7 μm; Bio-Rad), 1 μg of DNA, 50 μL of 2.5 M sterile CaCl_2_ (Sigma), and 20 μL of 0.1 M spermidine (Sigma). The mixture was vortexed for 10 min, centrifuged, and washed twice with 150 μL of absolute ethanol. The tungsten beads carrying the DNA were resuspended in absolute ethanol and distributed on macrocarrier disks. Bombardment of the plates by a biolistic transformation apparatus (Bio-Rad) was performed according to the manufacturer’s instructions, with a helium gas pressure of approximately 1,200 lb/in^2^ under 27 mm Hg. The plates were incubated for 24 h at 30°C, and the cells were then washed, transferred to YPD plates supplemented with hygromycin B or neomycin, and incubated at 30°C until colonies appeared on the agar surface. Mutants were screened by fluorescence microscopy. Genomic DNA from the selected transformants was used as a PCR template to confirm the presence of the fluorescence cassette on the genome (see Fig. S3 in the supplemental material). Multiplex PCR was used to identify if the cassettes were inserted into the safe haven region (Fig. S5), according to methods described previously by Arras et al. (37).

### Western blot analysis.

Each strain was grown as described above, washed, and resuspended in a solution containing 50 mM Tris-HCl (pH 8.8) with 1.25% Tween 20, 100 mM phenylmethylsulfonyl fluoride, a protease inhibitor cocktail (Roche), and glass beads. Cells were lysed and homogenized using a Precellys 24 device (5,500 rpm; 3 cycles of 15 s) (Bertin). Cell debris was removed by centrifugation, and the total protein concentration was determined by a Bradford assay (86). Ten micrograms of proteins from each sample was loaded onto a 12% SDS-PAGE gel and transferred to a polyvinylidene difluoride (PVDF) membrane in Tris-buffered saline–0.1% Tween 20 (TBST) buffer with a prestained protein ladder (S2600 TrueColor 9 to 245 kDa; Sinapse Inc., Brazil). Membranes were blocked with TBST containing 3% bovine serum albumin (BSA) and then incubated with the primary antibody rabbit IgG anti-GFP (catalog number A21312; Invitrogen) (1:2,000) or rabbit IgG antiactin (catalog number A2066; Sigma) (1:100) overnight at 4°C under agitation. Next, after washing steps, the membranes were incubated with goat anti-rabbit IgG–horseradish peroxidase (HRP) (catalog number ab6721; Abcam) (1:10,000) for 1 h at room temperature. The membranes were washed, incubated with a luminol-based chemiluminescent substrate (Abcam), and imaged using an AI600 device (GE Healthcare).

### Fluorescence microscopy and staining.

C. neoformans WT and mutant strains were evaluated for fluorescence emission and the proper cellular compartment localization of histone H3 or Pab, tagged with GFP or mCherry, using a Zeiss Axio Observer Z1 inverted phase-contrast fluorescence microscope (Carl Zeiss) equipped with a 63× 1.4-numerical-aperture (NA) oil immersion objective, the high efficiency (HE) filters 38 HE (green) and 63 HE (red), and differential interference contrast (DIC). Images were captured using ZEN lite 2012 SP2 software (Carl Zeiss). Viable but nonculturable yeast cells were imaged using an Evos fluorescence microscope (Thermo Fisher) with 357/44-nm (4′,6-diamidino-2-phenylindole [DAPI]) and 470/22-nm (GFP) LED lights coupled to 447/60- and 510/42-nm emission filters, respectively.

For the visualization of nuclei, cells were stained with NucBlue live-cell nuclear stain (Thermo Fisher) for 5 min. For viability analysis, cells were incubated with LIVE/DEAD violet dye (LVD) (excitation/emission at 405/450 nm) (Thermo Fisher) for 20 min at room temperature with protection from light. As a control, dead cells were used after heat inactivation for 1 h at 70°C (heat killed [HK]). Cells were washed twice prior to analysis.

### Flow cytometry analysis.

The fluorescence intensity produced by each strain was determined by flow cytometry using a FACSVerse instrument (BD). Each strain was grown as described above, washed, and resuspended (5 × 10^5^ cells/mL) in phosphate-buffered saline (PBS). Measurements were acquired using 488- and 633-nm excitation lasers paired with 527/32- and 660/10-nm emission filters for GFP and mCherry, respectively. Data were analyzed using FlowJo v.10 software. After the exclusion of cell debris and doublets, the median fluorescence intensity (MFI) obtained for each strain was normalized to that of the WT.

### Evaluation of phenotypes and virulence determinants.

C. neoformans strains were tested for virulence determinant expression and phenotypic traits under the following conditions: osmotic (1.5 M NaCl), cell wall (0.5% Congo red and 0.05% SDS), and oxidative (1 mM H_2_O_2_ and 25 and 35 μM menadione) stresses and high temperature (37°C). Each strain was grown overnight at 30°C in liquid YPD medium at 150 rpm, washed with a saline solution, and serially diluted from 10^7^ to 10^3^ cells/mL. A total of 5 μL of each dilution was spotted onto YPD agar supplemented with the respective stressor agents. Capsule production was examined after growth as described previously (87), using India ink counterstaining. The cells were photographed using a Zeiss Axio Observer Z1 microscope (Carl Zeiss), and the capsule thickness was measured using ZEN lite 2012 SP2 software (Carl Zeiss). Melanin synthesis was visualized in Niger seed agar, whereas urease and phospholipase activities were evidenced in Christiansen’s urea agar and plates prepared with an egg emulsion (88), respectively, after 5 μL of a suspension of 2 × 10^7^ cells/mL of each strain was spotted. To observe mating filaments, each transformant was crossed to KN99*a* (MAT**a**). Cell cultures were grown overnight (30°C under agitation at 150 rpm), washed, resuspended, and adjusted to 2 × 10^7^ cells/mL in a saline solution. Equal amounts of **a** and α strains were mixed, and 5 μL of each mixture was spotted onto agar filament plates (6.7 g/L of yeast nitrogen base [YNB] without amino acids and without ammonium sulfate, 0.5% glucose, and 4% agar at pH 5.0) or onto MS (Murashige-Skoog) agar (89) and then incubated at room temperature in the dark for 1 week or 10 days, respectively. The mating assays on filament agar were photographed using a stereomicroscope (Leica), and the filaments were scraped from the agar surface and deposited onto slides for fluorescence microscopy visualization. Each experiment was independently repeated three times. For microscopic inspections of the sexual reproductive structures (basidia and basidiospores), the MS agar plates from a single experiment were photographed at a ×200 magnification using SporePlay+ equipment (Singer Instruments).

### C. neoformans growth assays.

The growth kinetics of the WT and the fluorescent strains were evaluated at 30°C in liquid YPD medium. Each culture was adjusted to 5 × 10^4^ cells per mL, and 200 μL/well was added to a 96-well plate. The plate was incubated under continuous agitation, and the optical density was measured every 30 min at 600 nm using an Eon BioTek spectrophotometer for 96 h. For latency (lag- or λ-phase) assessment, each strain was cultured as described above but at 10^4^ cells/mL. Analysis was performed using Gen5 Data Analysis software (BioTek). Growth rate analysis was conducted using GraphPad Prism v.7.0 software according to methods described previously by Hommel et al. (21).

### Ethics statement.

All procedures involving animals and their care were approved by the Animal Ethics Committee of the University of Brasília (UnBDoc number 66729/2016) and conducted in accordance with Brazilian Council for the Control of Animal Experimentation (CONCEA) guidelines.

### Generation of bone marrow-derived macrophages.

Bone marrow-derived macrophages (BMMs) were obtained by using a previously described method (90). Briefly, murine femurs and tibias from 8- to 10-week-old C57BL/6 mice were dissected and flushed with cold RPMI 1640 (Sigma-Aldrich) to release bone marrow cells. Cells were centrifuged (300 × *g* for 5 min at 4°C); resuspended in red blood cell lysis buffer (Sigma-Aldrich); washed; plated at 2 × 10^6^ cells/petri dish in 10 mL of RPMI 1640 medium (Sigma-Aldrich) containing 20% heat-inactivated fetal bovine serum (FBS), 30% L929 cell-conditioned medium, and 25 μg/mL of gentamicin; and incubated in a 5% CO_2_ atmosphere at 37°C. On day 4, medium was replaced with 10 mL of fresh complete medium. On day 7, macrophage monolayers were washed with fresh medium and detached using TrypLE Express (Gibco). Next, cells were washed, resuspended in experimental medium composed of RPMI 1640 supplemented with 10% FBS, and used for experiments.

### Assays of phagocytosis by murine macrophages.

The phagocytosis of C. neoformans strains was evaluated by 3 techniques: (i) common light microscopy and panoptic staining (LB, Brazil), (ii) fluorescence microscopy, and (iii) flow cytometry. Prior to the yeast-macrophage interaction, macrophages were seeded at 5 × 10^4^ cells per well in a 96-well plate for surface adhesion. In parallel, C. neoformans cells were opsonized with 10 μg/mL of the anti-GXM monoclonal antibody 18B7 (a donation from Arturo Casadevall) for 20 min and used for infection of macrophages at a multiplicity of infection (MOI) of 5. After 2 h of infection, wells were washed to remove nonphagocytized yeast cells, and the analysis proceeded according to the techniques described below.

For light microscopy, panoptic staining was used for all strains. For the fluorescence control, the WT strain was labeled with the fluorophore fluorescein isothiocyanate (FITC). Yeast cells were incubated for 10 min with 0.5 mg/mL of FITC (Sigma-Aldrich) diluted in PBS and then washed, according to methods described previously (91). After staining/labeling, each sample was opsonized and used for the interaction assay. After 2 h of infection, wells were washed to remove nonphagocytized yeast cells, and the cells were incubated with DAPI (0.5 μg/mL), fixed with 4% paraformaldehyde for 20 min, and further analyzed by fluorescence microscopy at a ×400 magnification for phagocytosis evaluation. The percentage of phagocytosis was calculated according to methods described previously by Roilides et al., by dividing the number of BMMs with ≥1 phagocytized yeast cell by the total BMMs observed, multiplied by 100 (92). In addition, the phagocytic index was obtained by calculating the average number of internalized yeast cells per phagocyte.

For flow cytometry analysis, macrophages (1.2 × 10^6^) were seeded into petri dishes with a 5-mm diameter. C. neoformans coincubation with macrophages, including the FITC-labeled WT, was performed as described above. After incubation, each macrophage monolayer was washed with warm PBS, detached, and labeled with APC-conjugated anti-CD11b antibody in PBS plus 2% FBS for the identification of macrophages. An isotypic antibody was used as a control. Subsequently, each cell suspension was washed and subjected to flow cytometry. The percentage of phagocytosis was obtained after gating CD11b^+^ events (macrophages) followed by percentage calculations of the number of events positive for FITC/GFP (representative of macrophages containing internalized fluorescent yeast cells) in relation to the number of total events acquired by the BD FACSVerse cytometer, using FlowJo v.10 software.

### Killing assay using activated murine macrophages.

Before the interaction with yeast cells, BMMs were activated with 1 μg/mL of lipopolysaccharide (LPS) (Sigma-Aldrich) and 20 ng/mL of interferon gamma (IFN-γ) (Immunotools) for 3 h. In parallel, C. neoformans cells were opsonized as described above and used for infection of BMMs (MOI of 2). After 2 h of infection, PBS-washed cell monolayers were lysed with a 0.05% SDS solution, and the resulting fungal suspension was serially diluted in PBS and plated onto Sabouraud dextrose agar (SDA) medium (10 g/L peptone, 40 g/L glucose, 15 g/L agar [pH 5.6]) at 30°C for CFU determination. For 24-h CFU counting, after washing, infection was allowed to proceed for 24-h, and the assay was performed as described above. The percentage of fungal survival was calculated based on the 24-h/2-h CFU ratio (for each strain) multiplied by 100.

### *In vivo* virulence assays in Galleria mellonella.

For *in vivo* virulence assays, G. mellonella caterpillars in the last larval stage were kept in the dark at 30°C and used for infection. Each C. neoformans strain was grown and washed as described above and resuspended in PBS at 5 × 10^6^ yeast cells/mL. For each strain tested, 14 to 17 larvae per group were inoculated with 10 μL (containing 5 × 10^4^ cells) of the yeast suspension into the penultimate proleg using a Hamilton syringe and incubated at 37°C. An uninfected control group received only PBS. The larvae were examined daily after inoculation, and the scores of dead larvae were used to perform survival curves by applying Kaplan-Meier analysis using the log rank and Wilcoxon tests (93). After 5 days of infection, dead larval samples were flushed with 100 μL of PBS to recover larval hemolymph. The hemolymphs were further diluted 10-fold with PBS, and samples were analyzed using an Axiovert 100 fluorescence microscope (Carl Zeiss) to visualize the yeast cells and evaluate the maintenance of the green or red fluorescence emission. After the determination of the LT_50_, G. mellonella caterpillars were inoculated with the transformant strains and incubated as mentioned above for the assessment of the fungal burden by CFU counting. At the LT_50_, G. mellonella caterpillars (*n* = 5) were homogenized using a Precellys 24 bead-beating system (Bertin Technologies) with steel beads for 2 cycles of 15 s each at 4,000 rpm. Sample homogenates were serially diluted, plated onto YPD agar (pH 5.6) supplemented with 50 mg/L gentamicin (Gibco), and incubated at 30°C.

### Obtaining C. neoformans viable but nonculturable cells.

Viable but nonculturable (VBNC) C. neoformans cells were obtained as described previously by Hommel et al. (21). Briefly, cells (approximately 10^7^ cells) obtained from 2- to 5-day-old SDA plates were cultured in a T25 flask with a ventilated cap containing 10 mL of YPD liquid medium at 150 rpm at 30°C for 22 h. To ensure that the culture reached the stationary phase, 100 μL of this preculture was used as an inoculum in fresh medium subjected to an additional 22 h of incubation. To induce dormant yeast cells, this stationary-phase culture (STAT) was placed within a hermetically sealed plastic bag containing a hypoxic-atmosphere generator (<0.1% oxygen) (GENbag anaero; bioMérieux) for 7 days in the dark at 30°C in a static incubator.

Yeast culturability (i.e., the ability of a yeast cell to form a colony) was assessed by determining the number of CFU. Yeast cells were counted using a hemocytometer (Neubauer chamber), and the cell concentration was adjusted to 1 × 10^5^ cells/mL in PBS. One hundred microliters of this suspension was inoculated onto SDA plates in duplicates and incubated at 30°C for 5 days for CFU counting.

### Statistical analysis.

Statistical analyses and significance determinations were performed using GraphPad Prism v.7.0 software. Data were analyzed by one- or two-way analysis of variance (ANOVA), followed by Dunnett’s or Tukey’s posttest for comparisons to the WT control group or between the groups indicated, respectively. Two-tailed Student’s *t* test was used to compare two groups. *P* values of less than 0.05 were considered significant.

### Data availability.

The yeast strains generated in this paper are available upon request.
