# Supplementary material for: Engineered Fluorescent Strains of Cryptococcus neoformans: a Versatile Toolbox for Studies of Host-Pathogen Interactions and Fungal Biology, Including the Viable but Nonculturable State
Source: Microbiol Spectr. 2022 Aug 25;10(5):e01504-22. doi: 10.1128/spectrum.01504-22 (PMC9603711; doi:10.1128/spectrum.01504-22)
Supplement: Supplemental file 1 — Supplemental material. Download spectrum.01504-22-s0001.pdf, PDF file, 0.7 MB [file spectrum.01504-22-s0001.pdf]

Castro, R.J.A.d. Supplemental material

Table S1. Oligonucleotides used.

Name	Sequence (5’ > 3’)	Fragment size	Reference
**LF498**	CTAGGATCCATGGCCCGAACGAAGCAGACC (BamHI)	0.85 kb	This study
**LF499**	CTAGGATCCGATAGTCGCCATCAGCCAGTC (BamHI)		This study
**LF502**	CTAGGATCCATGTCCGTCGAAACCGCTACAT (BamHI)	2.94 kb	This study
**LF503**	CTAGGATCCGGGTGTTGAGGCCTGTGAGTA (BamHI)		This study
**LF512**	GGTCGACGGTATCGATGTGTGGAATTGTGAGCGGATA (ClaI)	HG/ HM – 2.30 kb<br>PG/ PM – 4.40 kb<br>GØ – 1.50 kb<br>MØ – 1.50 kb	This study
**LF513**	TG**GAGCTC**CACCGCGGCTTCATGGCTCCTTGTCTCTG (**SacI** - SacII)		This study
**LF566**	CTGACCAGTCTTCCTCAA	PG/ PM – 1.32 kb (In combination with LF559)	This study
**LF501**	CTACCATGGAGACCTCTCGCCTCGGAGAC	HG/ HM – 1.46 kb (In combination with LF559)	This study
**LF559**	GGAAGATATCTCAGCCAGTA	-	This study
**UQ1768**	TCAGCAACGCCGTTGAATCCT	-	Arras et al. (2015)
**UQ2962**	GGGTATGCCACAGATGCAGAT	-	Arras et al. (2015)
**UQ2963**	TTGGATCCTCAATTGTCTCCT	-	Arras et al. (2015)
**UQ3348**	ACTGGTGAGTACTCAACCAAG	-	Arras et al. (2015)

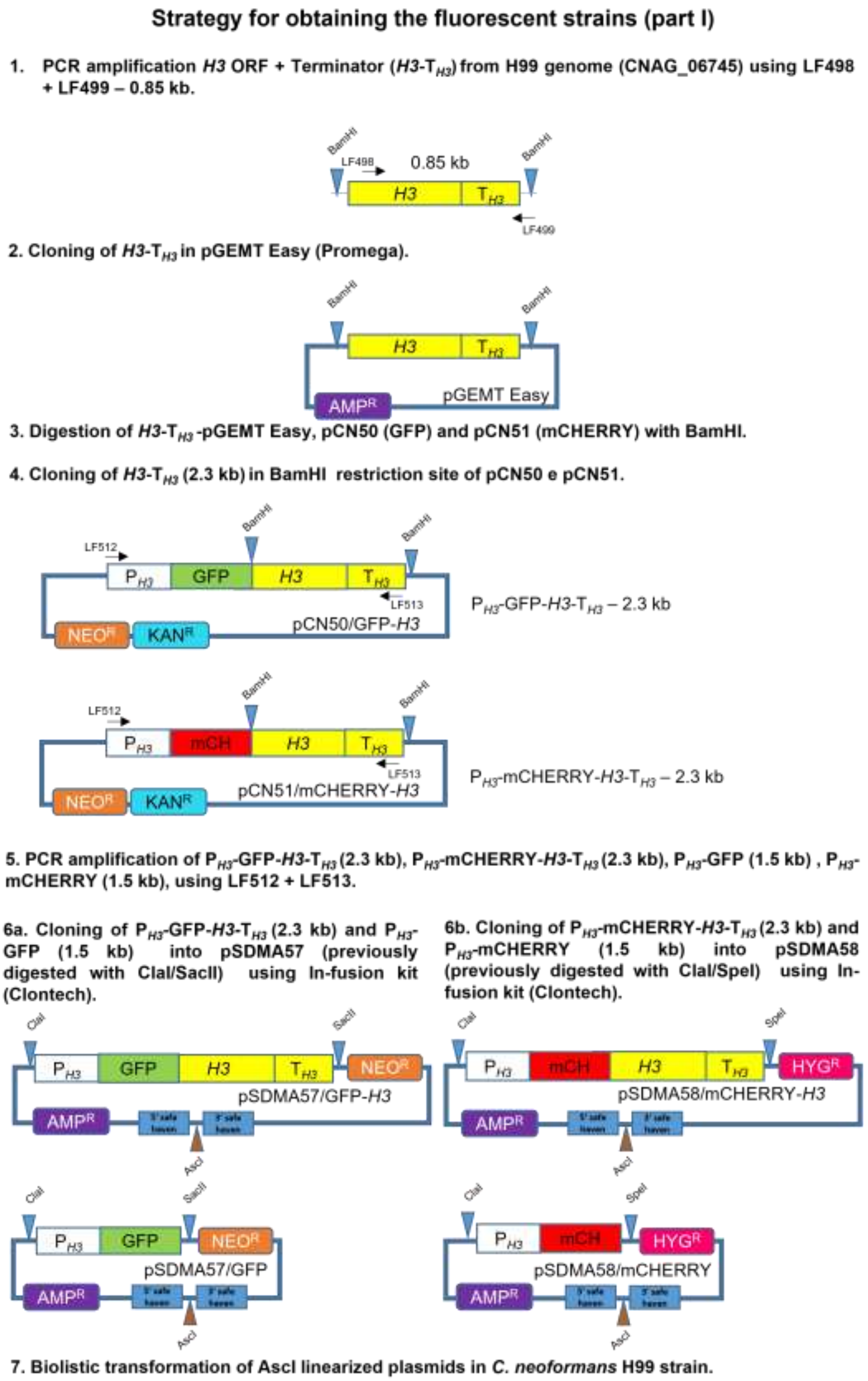

Fig S1. Strategy of the cassette construction for obtaining H3-GFP, H3-mCherry, ø GFP and ø Cherry strains.

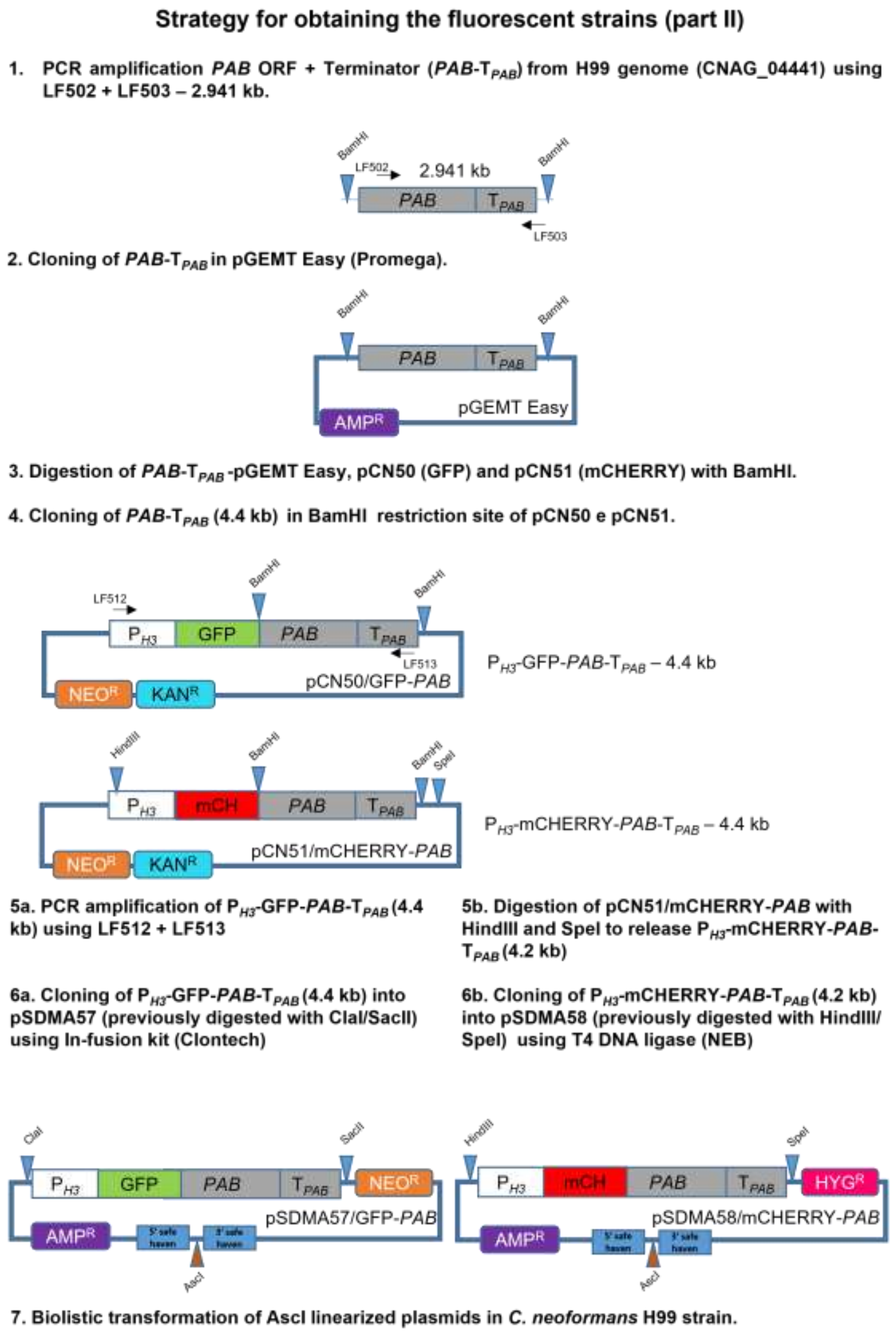

Fig S2. Strategy of the cassette construction for obtaining PAB-GFP and PAB-mCherry strains.

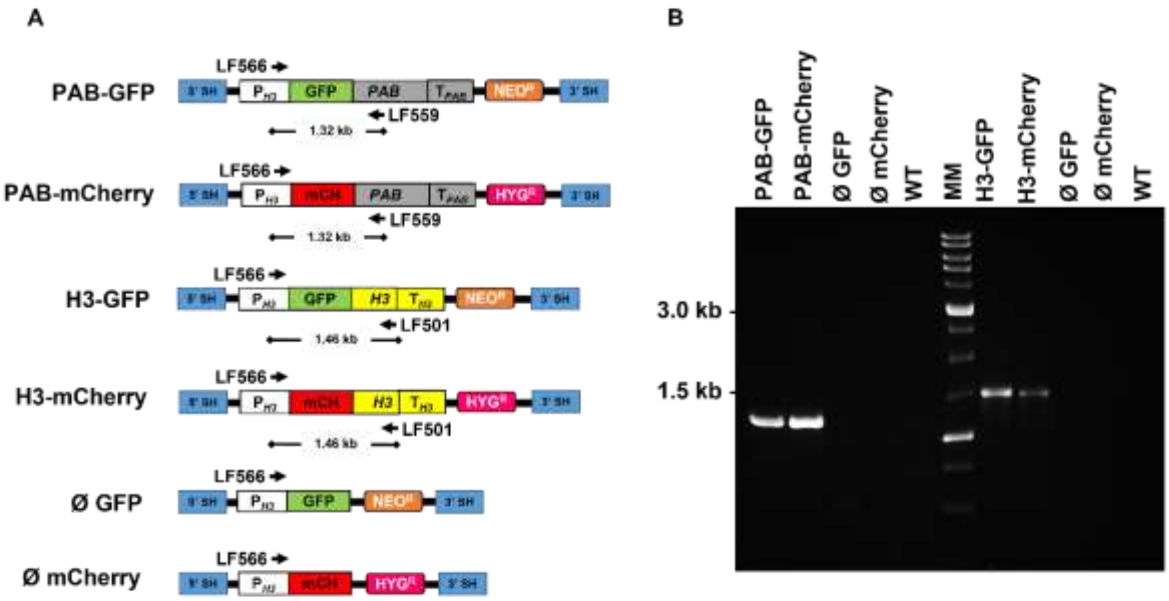

Fig S3. Detection of PAB-GFP, PAB-mCherry, H3-GFP and H3-mCherry fusion cassettes on fluorescent strains genome by PCR. (A) A schematic representation of the cassettes inserted into the genome of the fluorescent strains. Oligonucleotides positions and sizes of the amplicons are indicated. SH: Safe haven. (B) Amplification of the DNA fragments related to fusions PAB-GFP, PAB-mCherry, HIS3-GFP and HIS3-mCherry from the fluorescent strains. Oligonucleotides LF566 and LF559 were used to amplify 1.32 kb of PAB-fusions while LF566 and LF501 amplified a 1.46 kb region of HIS3- fusion cassettes. The negative controls were: gDNA isolated from WT, ø GFP and ø mCherry strains. MM: Molecular Marker (1 kb Ladder, Kasvi).

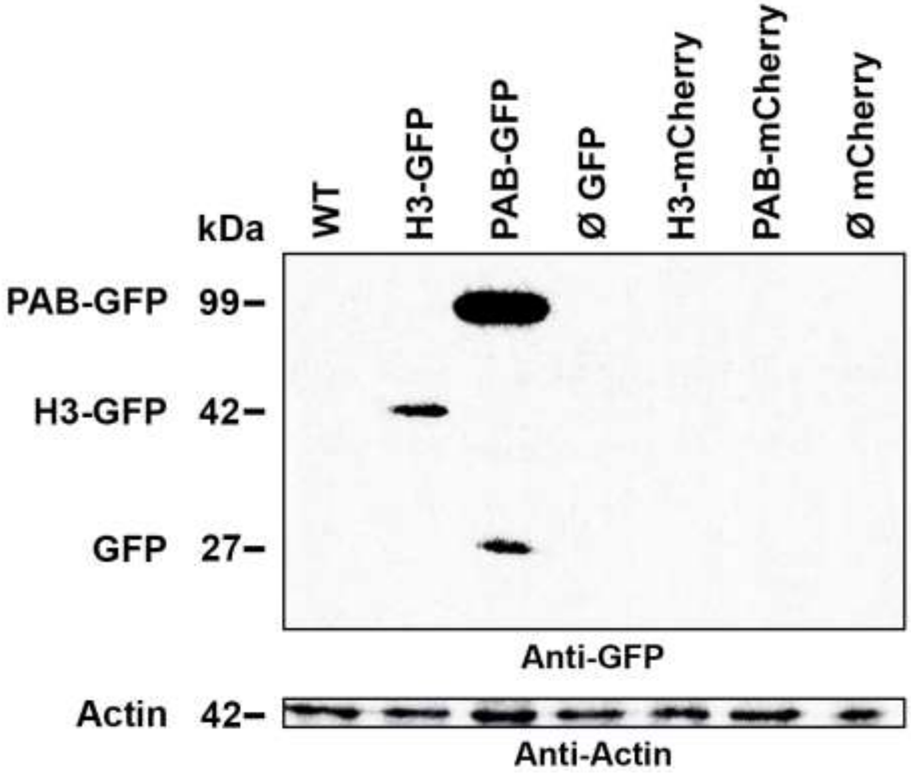

Fig S4. Detection of GFP-tagged proteins on transformants by western blot analysis. Detection of His3- or Pab1-GFP fusion protein in *C. neoformans* transformant strains.

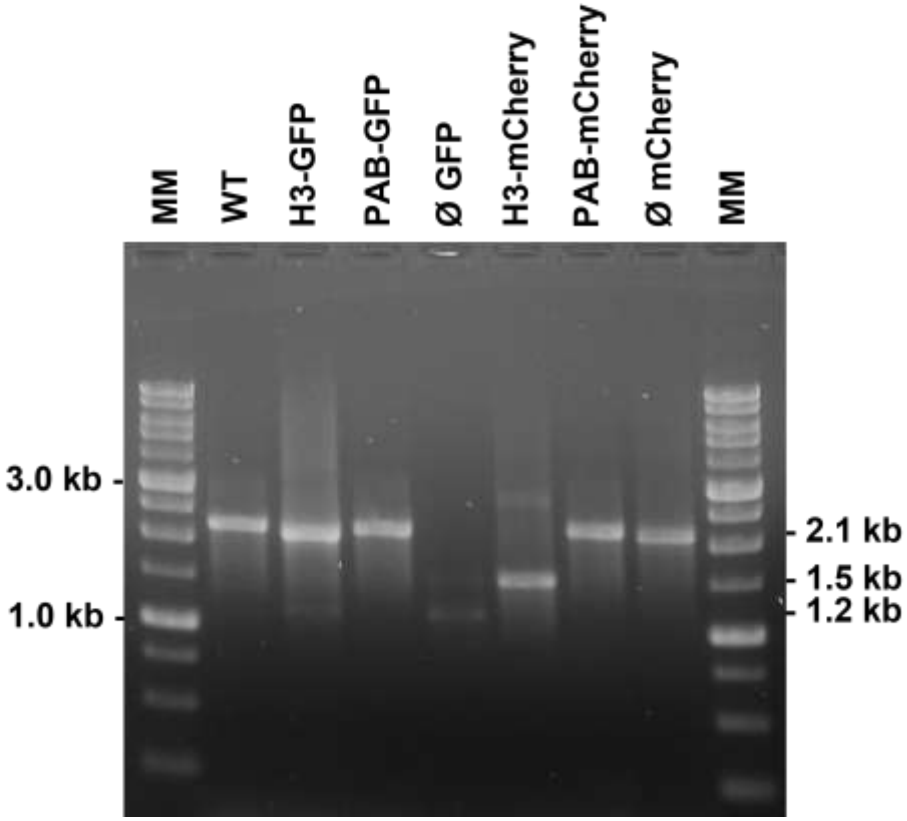

Fig S5. Multiplex PCR to check whether the cassettes were inserted in Safe haven (SH) genomic region. Genomic DNA from the selected mutants and H99 was extract and used as template accordingly to Arras et al., 2015. A 2.17 kb amplicon is indicative of random genomic integration, while two fragments of 1.5 and 1.2 kb correspond to integration of the cassette on SH region. Only ø GFP mutant had the cassette inserted on SH region, the others (H3-GFP, PAB-GFP, PAB-mCherry, and ø mCherry) had a random integration into the genome, while H3-mCherry presented two unexpected amplicons of 3 and 1.5 kb which are suggestive of a genomic rearrangement of SH region.

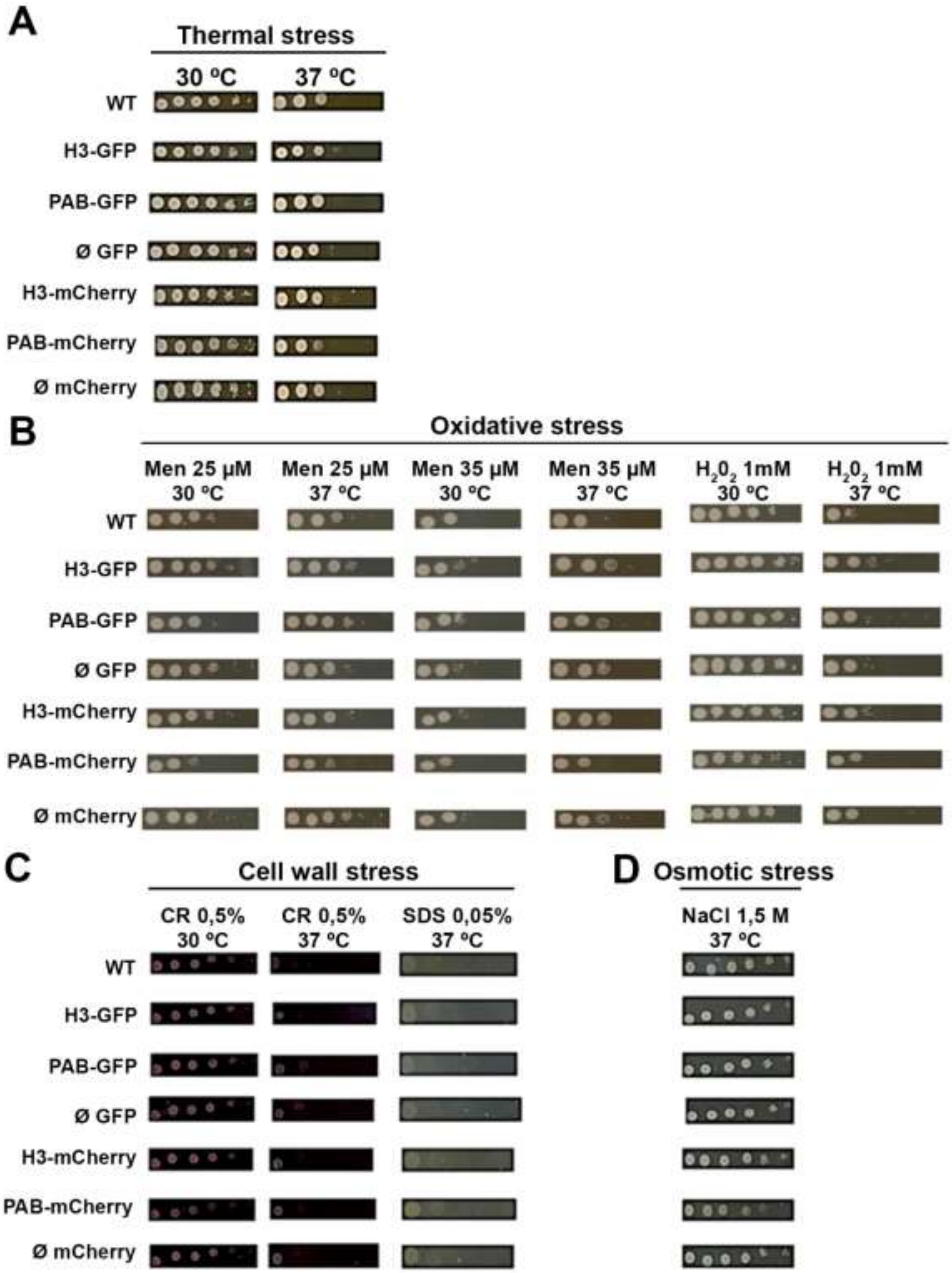

Fig S6. The transformant strains exhibit no alterations in stress-tolerance response in comparison to the parental strain. Yeast suspensions were subjected to serial dilutions (2 × 10^7 to 2 × 10^2 cells/mL) and plated (5 µL) onto YPD agar plate and incubated at 30ºC and

37ºC (A), or YPD containing the indicated concentrations of menadione (Men) or H_2O_2 (B), Congo Red (CR) or SDS (C) or NaCl (D).

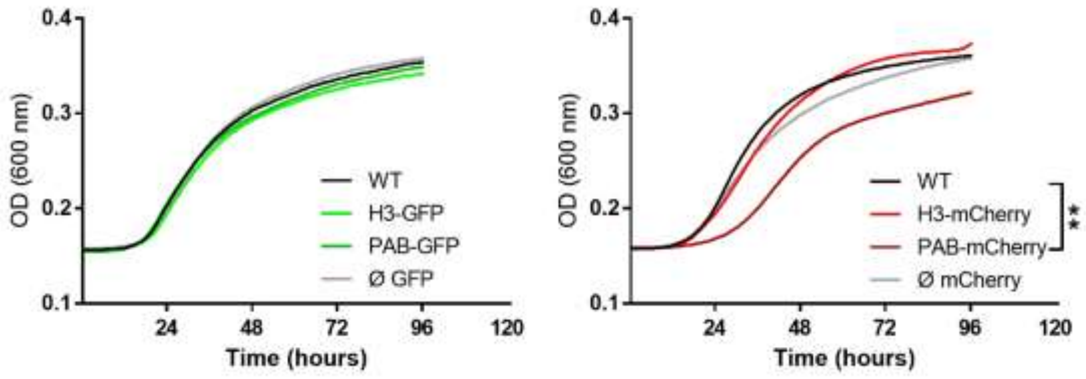

Fig S7. Transformant strains, except PAB-mCherry, exhibit growth kinetics similar to the parental strain. Yeasts (10^4/mL) were grown in liquid YPD medium at 30ºC for growth analysis. Optical density at 600 nm (OD600) was monitored over time. **p <0.01, compared to WT.

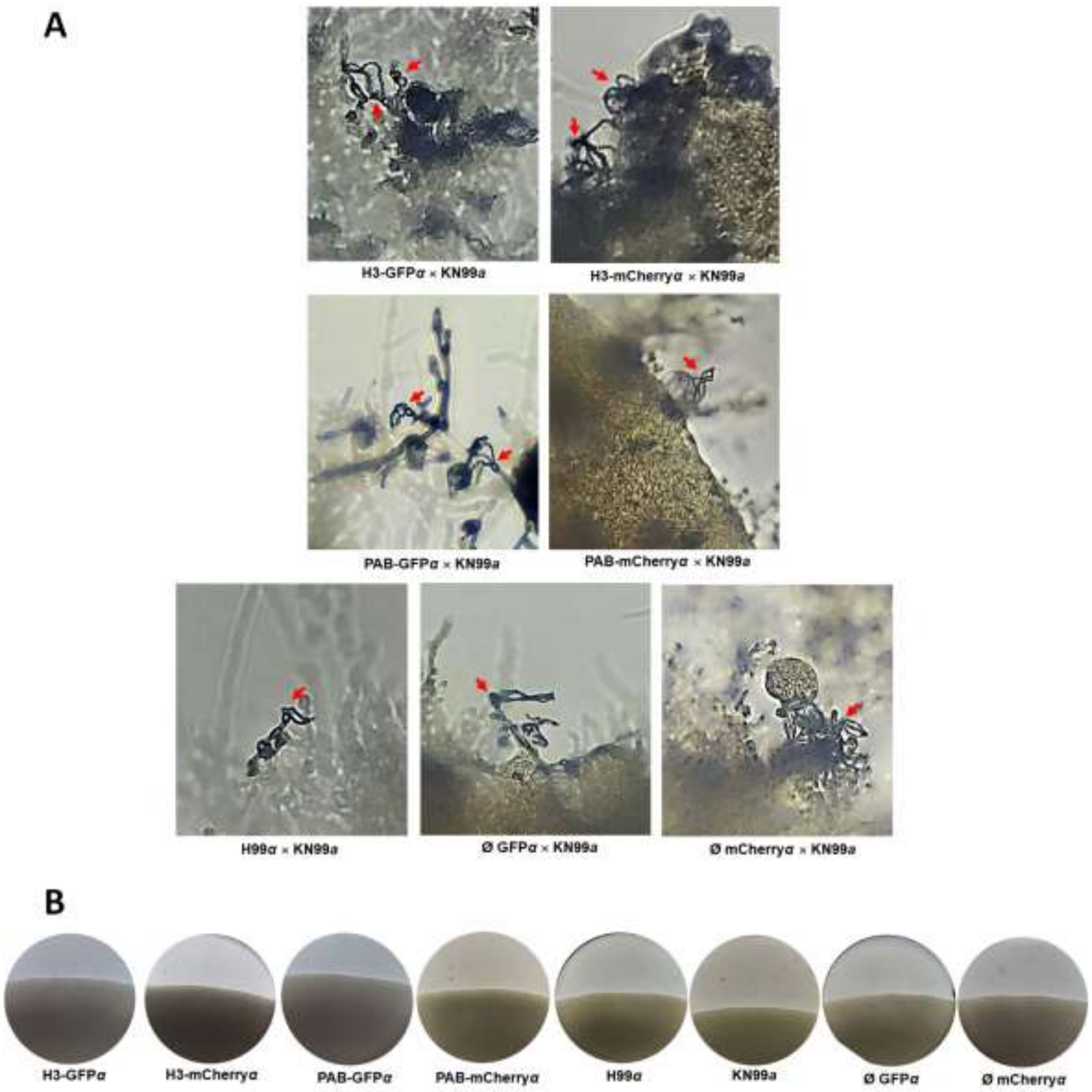

Fig S8. Fluorescent strains produce mating structures: basidium and basidiospores chains. (A) The mating structures: basidium and basidiospores chains were visualized in all crossings after 10 days of co-culture of equal numbers of cells for the fluorescent strains (MAT*α*) and KN99 (MAT*a*) in MS agar medium and they are indicated by red arrows. (B) Spots of individual strains in the absence of the sexual partner did not display filaments after 10 days of incubation in MS medium at room temperature. H99*α* and KN99*a* are wild-type partners control strains. Magnification of the images were ×200. MS: Murashige and Skoog (MS) agar medium.
